# Gas-propelled microneedles for precision transdermal therapeutics

**DOI:** 10.1016/j.bioactmat.2026.08.059

**Published:** 2026-09-03

**Authors:** Zhicheng Xiao, Fan Jia, Wentao Wu, Hoiian Ieong, Zeshi Jiang, Siyuan Peng, Chuanbin Wu, Wenhao Wang, Xin Pan, Zhengwei Huang

**Affiliations:** a School of Pharmaceutical Science, Sun Yat-Sen University, Guangzhou, 510006, China; b State Key Laboratory of Bioactive Molecules and Druggability Assessment, Guangdong Basic Research Center of Excellence for Natural Bioactive Molecules and Discovery of Innovative Drugs, Jinan University, China; c Jiangmen Wuyi Hospital of Traditional Chinese Medicine, Affiliated Jiangmen Traditional Chinese Medicine Hospital of Jinan University, Jiangmen, 529031, China; d Key Laboratory of Drug-Targeting and Drug Delivery System of the Education Ministry, Sichuan University, Chengdu, 610041, China

**Keywords:** Gas-propelled microneedles, Active transdermal delivery, Gas therapy, Microenvironment modulation, Controlled gas release, Biomedical applications

## Abstract

Transdermal drug delivery is an appealing approach because it is easy to administer, bypasses first-pass metabolism, and utilizes an extensive surface area of the skin. Microneedles, as an emerging transdermal drug delivery system, offer minimal invasiveness, painlessness, and targeted *in*-*situ* treatment. However, the existing microneedle technologies rely on passive diffusion, which can result in unpredictable drug penetration profiles. To enhance the active deep penetration and diffusion of cargoes of the microneedles, gas-propelled microneedles have gradually gained attention from researchers. Gas components can improve transdermal drug delivery efficiency by enhancing drug penetration via pressure effects and bubble generation. Meanwhile, therapeutic gases themselves may function as bioactive components, providing synergistic therapeutic benefits in addition to drug delivery. Hence, Gas-propelled microneedles are promising drug delivery platforms. This review first introduces the structure of the skin and the mechanisms underlying transdermal permeation, followed by a comprehensive overview of recent advances in gas-propelled microneedles for enhancing transdermal drug delivery. We outlined the optimal application strategies based on the distinct properties of different gas-propelled microneedles. It also underscored optimal design strategies for gas-propelled microneedles, assisting formulators in making well-informed decisions. Ultimately, we discussed the current shortcomings of gas-propelled microneedles and future development directions.

## Highlights

• Therapeutic and permeation-enhancing functions of diverse medical gases are systematically compared.
• Rational design strategies are proposed from materials, structures, gases, and bioactive carriers.
• Perspectives on controllability, biosafety, mathematical modeling, and clinical translation are discussed.

## Graphical abstract

**Figure:**
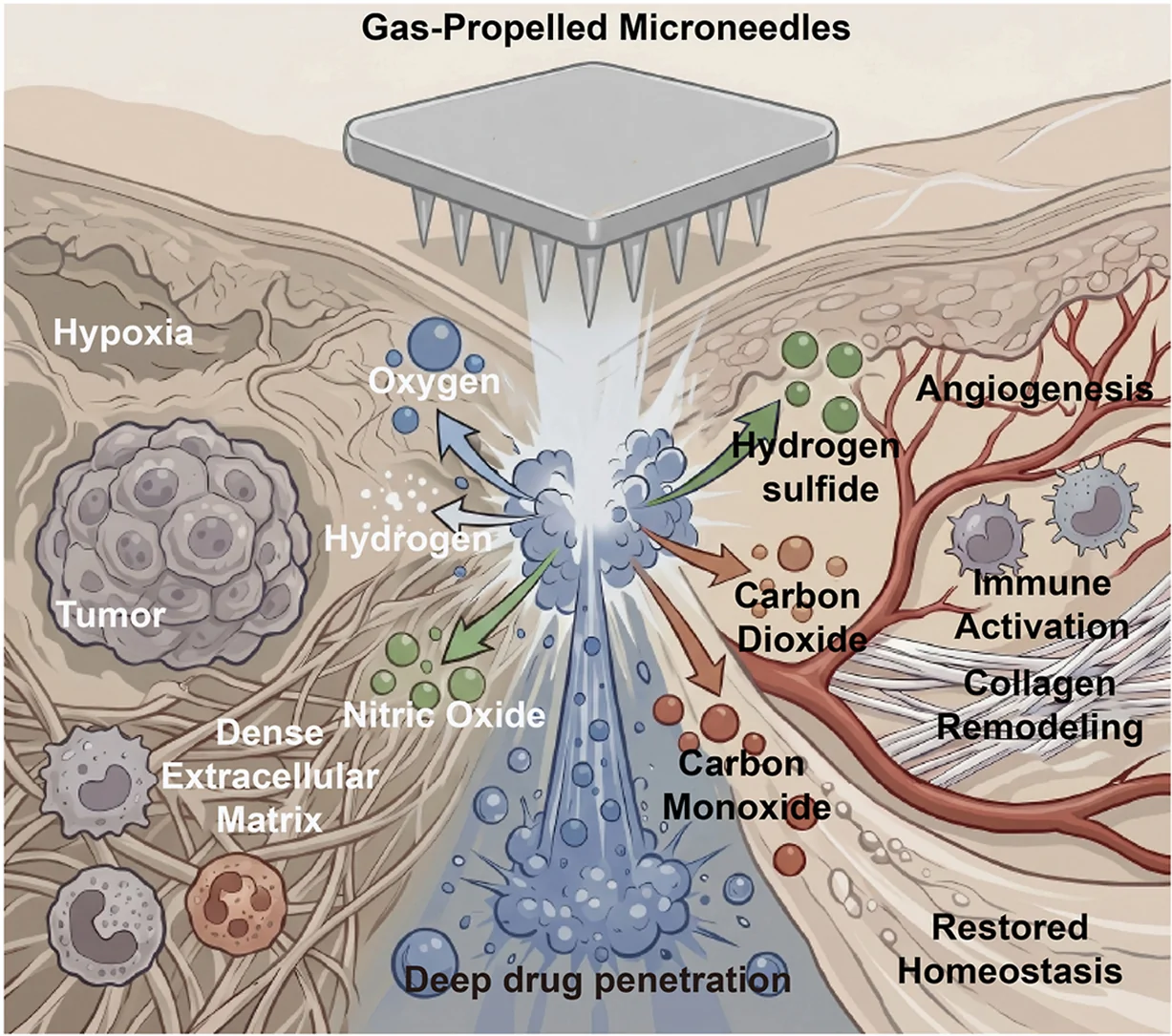

## 1 Introduction

Microneedle transdermal drug delivery is a minimally invasive drug delivery method that utilizes tiny needles, usually measuring between 100 and 1000 μm in length, to penetrate the outermost layer of the skin. Compared to traditional needles, microneedle transdermal delivery significantly reduces pain and discomfort [1]. It enhances drug absorption and leads to a faster onset of action while being applicable to various therapeutics, including chemical, natural, and biological drugs [2,3]. This technology improves patient compliance and lowers the risk of infection, making it an effective and comfortable option for drug delivery [4,5]. For example, microneedles have been approved by the Food and Drug Administration (FDA) for delivery of vaccines and pharmaceuticals through the epidermis in clinical settings [[6], [7], [8]]. Nevertheless, once the microneedles are inserted into the skin and the drug is delivered into the body, its absorption relies primarily on passive diffusion [9]. The defect limits the current applications of traditional dissolving microneedles, particularly in scenarios involving thickened skin or necessitating deep tissue penetration.

To tackle this challenge, various external stimuli have been utilized to enhance drug permeation through the epidermis by altering the skin's barrier properties. These stimuli include electroporation [10,11], ultrasound [12], light [13], and temperature [14]. However, these methods require device regulation, which can be inconvenient, complicated, and costly, making them less suitable for diverse settings, including home use. Additionally, some researchers are focusing on modifying the drugs delivered by microneedles. For instance, incorporating penetration enhancers with the drugs aims to regulate drug diffusion within the body after release [15]. However, introducing these additional modules or specific drug modifications inevitably adds more components, raising safety concerns and hindering broader application [16].

Therefore, developing a simpler and more efficient strategy to enhance drug penetration and delivery remains highly desirable. Gas-propelled microneedles have emerged as a promising method to enhance drug penetration and diffusion, compared with pristine microneedles [17]. Utilizing various gases, such as hydrogen, oxygen, and nitric oxide, as physical propellants to accelerate drug release can empower drugs with active transport and diffusion capabilities, thereby maximizing their therapeutic effects. Moreover, the unique properties of these gases allow them to be used as therapeutic components in conjunction with drug delivery [[18], [19], [20]]. For instance, Zhang et al. utilized citric acid and potassium carbonate (K_2_CO_3_) as gas pressure initiators, generating Carbon dioxide (CO_2_) through an acid-base reaction to drive the permeation of rivastigmine into the deeper layers of the skin [21]. Liu et al. developed a photosensitizer-loaded microneedle patch with sodium percarbonate (Na_2_CO_4_) for a deeper and faster transdermal delivery of photosensitizer and improved photodynamic therapy by embedding Oxygen (O_2_) propellant into microneedle [18]. However, although several studies have been reported, there remains a lack of systematic review, critical analysis, and comprehensive discussion in this field, thereby impeding the rational design and optimization of gas-propelled microneedle systems. In addition, there is currently no comprehensive review that systematically integrates gas generation mechanisms, propulsion principles, gas-mediated therapeutic functions, engineering design strategies, and clinical translation within a unified framework for gas-propelled microneedles. Moreover, the interplay between the physical propulsive role of gases and their intrinsic biological activities has not been critically discussed, making it difficult to establish rational design principles for different disease indications.

**Fig. 1:**
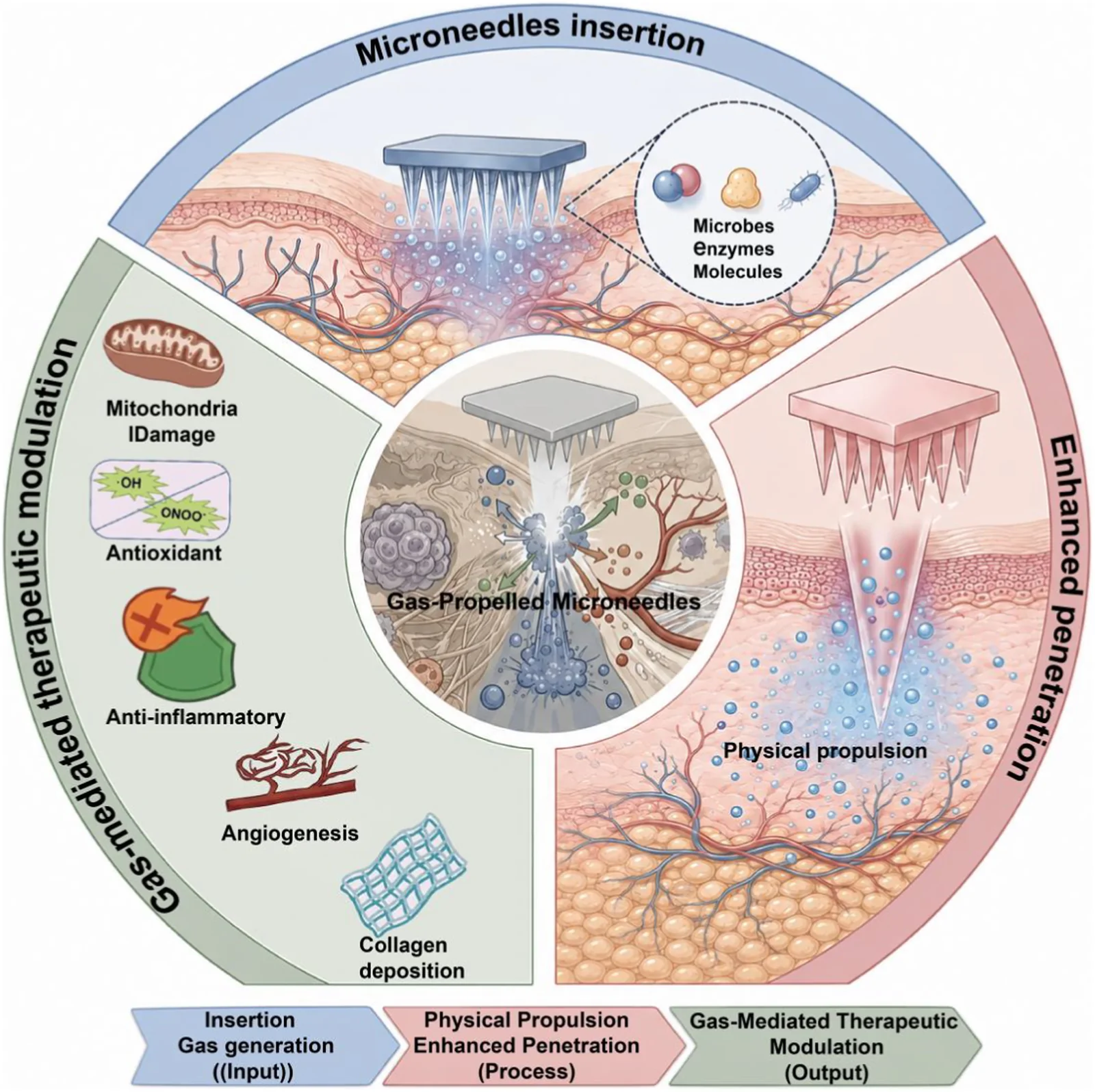
Schematic illustration of gas-propelled microneedles for gas delivery and the underlying therapeutic mechanisms.

The overarching goal of this review is to present an informative summary of the use of gas-propelled microneedles as a potential delivery platform of payloads to elevate penetration and diffusion behaviors, which is of great clinical importance. We first provided a brief overview of the skin structure and transdermal permeation process of the drug. Then, we discussed the design strategies that have been implemented and investigated to achieve deep drug penetration. Afterward, we emphasized the optimal design strategies of different gas propellants in various scenarios, enabling formulators to make more informed and critical decisions when manufacturing gas-propelled microneedles. Ultimately, we summarized the current shortcomings of gas-propelled microneedles and future development directions. We believe that such a review would act as a valuable guidance for the future design and development of gas-propelled microneedle platforms (Fig. 1).

## 2 Skin barrier and active transdermal delivery strategies

**Fig. 2:**
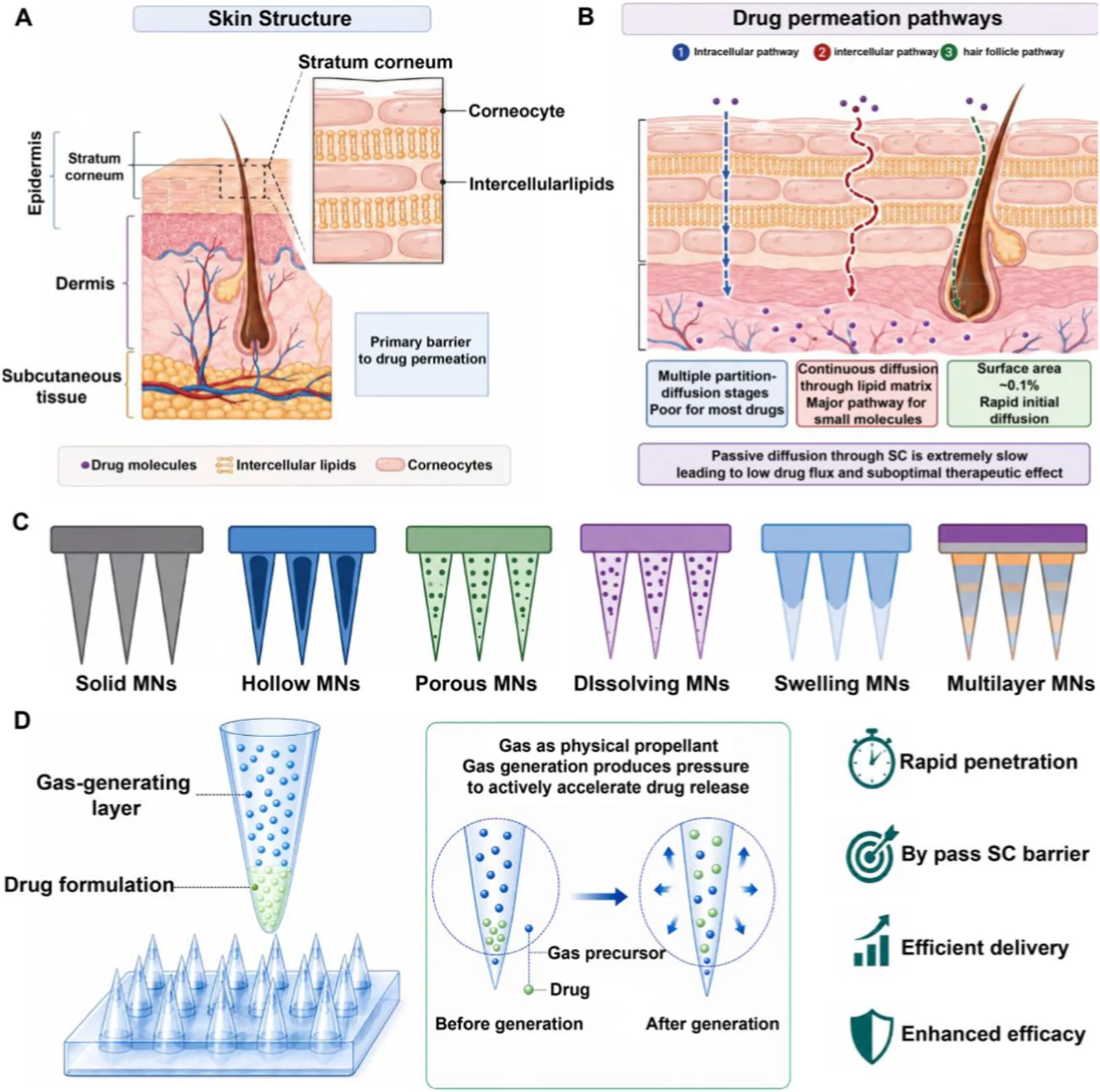
**Schematic illustration of skin structure and gas-propelled microneedle delivery.** (A) Structure of the skin, showing the stratum corneum (SC), epidermis, dermis, and subcutaneous tissue. The SC, composed of corneocytes and intercellular lipids, acts as the main barrier to drug penetration. (B) Three main drug permeation pathways across the skin: intracellular, intercellular, and hair follicle pathways. Passive diffusion through the SC is slow and limits drug delivery efficiency. (C) Different types of microneedles, including solid, hollow, porous, dissolving, swelling, and multilayer microneedles. (D) Gas-propelled microneedle system. In situ gas generation creates pressure to actively accelerate drug release, enhance penetration, bypass the SC barrier, and improve therapeutic efficacy.

Skin may be a major barrier to the transdermal delivery of drugs. Skin is the largest organ of the human body, composed of three main layers: the epidermis, dermis, and subcutaneous tissue [22]. It serves as a protective barrier and interfaces directly with the external environment (Fig. 2A). Among these, the stratum corneum (SC) of the epidermis serves as the principal barrier. The SC exhibits a typical “brick-and-mortar” structure, in which corneocytes are embedded within a highly ordered intercellular lipid matrix, effectively restricting the penetration of most exogenous molecules.

Drug molecules can cross the skin through three main pathways: intracellular (transcellular), intercellular, and appendageal (hair follicle) pathways (Fig. 2B). Although the intracellular pathway is theoretically feasible, it requires drugs to repeatedly partition between hydrophilic and hydrophobic domains, resulting in multiple diffusion barriers [23,24]. Consequently, most drugs preferentially diffuse through the intercellular lipid matrix [25]. The intercellular pathway enables continuous diffusion along lipid bilayers and is considered the dominant route for small-molecule drugs [[26], [27], [28]] Despite these pathways passive diffusion across the SC is inherently slow due to the highly organized lipid architecture and the physicochemical constraints of drug molecules, including molecular size, polarity, and solubility [29]. As a result, transdermal drug flux is typically low, often leading to suboptimal therapeutic outcomes [30]. Therefore, relying solely on passive diffusion for effective drug delivery is clearly insufficient. Nowadays, numerous strategies have been developed to enhance transdermal drug delivery, including electroporation, ultrasound, chemical penetration enhancers, and conventional passive microneedles. Each approach exhibits distinct advantages and limitations depending on the therapeutic scenario. Electroporation and ultrasound can markedly increase skin permeability by temporarily disrupting the stratum corneum and enhancing molecular transport. However, both techniques require dedicated external equipment, precise parameter control, and trained operation, which may increase treatment complexity and limit their applicability in home-based settings. Chemical penetration enhancers improve skin permeability by altering lipid organization or protein structures within the stratum corneum, yet prolonged or repeated application may cause skin irritation, barrier disruption, or unpredictable changes in drug absorption. To overcome these limitations, the technology of gas-propelled microneedles has emerged as a promising solution.

Microneedle technology has emerged as an effective strategy to overcome the SC barrier by physically creating microchannels that directly access the dermal layer [31]. Microneedles can be fabricated from materials such as silicon, metals, ceramics, glass, carbohydrates, and biodegradable polymers, and drugs may be incorporated in the form of solutions, particulates, or colloidal systems (Fig. 2C). Various microneedle designs-including solid, hollow, porous, dissolving, swelling, and multilayer microneedles-have been developed to achieve different delivery mechanisms [32,33]. Building upon conventional microneedle systems, gas-propelled microneedles introduce an active delivery mechanism. By incorporating a gas-generating component within the needle structure, *in situ* gas production generates localized pressure that actively drives drug release and accelerates diffusion into deeper skin layers. This propulsion effect not only enhances penetration efficiency and bypasses diffusion limitations within the SC, but also improves drug distribution and therapeutic efficacy (Fig. 2D). Specifically, gas-propelled microneedles establish transient pressure gradients within the confined tissue microenvironment. The rapid formation and expansion of gas bubbles generate mechanical forces that induce localized convective flow of interstitial fluid, thereby actively transporting therapeutic agents away from the microneedle tips and promoting their penetration into deeper tissues. This bubble-driven convection complements molecular diffusion, significantly enhancing both the transport rate and the spatial distribution of payloads. In addition, the transient mechanical stresses generated during gas evolution may further facilitate drug permeation by locally reducing diffusional resistance around the microchannels and transiently increasing tissue permeability without causing permanent structural damage.

Beyond serving as a physical propellant, the generated gas itself may also participate directly in therapy. Many gaseous mediators, including O_2_, H_2_, NO, CO, and H_2_S, possess intrinsic biological activities after release, such as alleviating hypoxia, scavenging reactive oxygen species, regulating inflammation, promoting angiogenesis, and modulating immune responses. Consequently, the generated gas is not merely a driving force for drug transport but may also function as an active therapeutic component depending on the disease indication. Therefore, a defining characteristic of gas-propelled microneedles is the dual functionality of the generated gas. On one hand, it acts as a physical propellant that enhances transdermal transport through localized pressure generation. On the other hand, many gaseous mediators, including O_2_, H_2_, NO, CO, and H_2_S, exert intrinsic biological activities after release, such as regulating oxidative stress, inflammation, angiogenesis, and tissue repair. The relative importance of these two functions is not universal but depends on the intended therapeutic application. For example, in chronic wound healing, the biological activity of therapeutic gases may contribute substantially to treatment efficacy, whereas in applications focused on improving transdermal delivery of biologics or macromolecules, propulsion efficiency may represent the primary design consideration. Therefore, future gas-propelled microneedle systems should be rationally optimized according to disease-specific therapeutic objectives by balancing propulsion performance and gas-mediated bioactivity.

## 3 Mainstream categories of gas-propelled microneedles

Effective gas therapy through exogenous physical triggers or endogenous disease-environment responses is an emerging treatment approach. This therapy involves gases that can directly alter disease states, such as O_2_, nitric oxide (NO), carbon monoxide (CO), hydrogen (H_2_), and hydrogen sulfide (H_2_S), as well as non-therapeutic gases like CO_2_. The use of microneedles serves a dual purpose: it protects gases and their precursors from premature leakage, enhancing their utilization in affected areas, while also facilitating the permeation of drugs within the microneedles, significantly improving the effective payload of the medication. In this process, the relationship between gases and microneedles is complementary [34,35].

When gas is combined with microneedles, it generates pressure application and bubble generation that drives drug molecules through the stratum corneum of the skin. This gas dynamics can disrupt the lipid structure of the stratum corneum, creating tiny channels that enhance drug permeability. Additionally, the expansion of the gas aids in improving drug distribution, facilitating its diffusion across the skin. Based on the gas-propelled permeation mechanism, focusing on the therapeutic effects of gases, the following section provides a detailed overview of microneedles combined with different gases (Table 1).

**Table 1:** Comparative overview of therapeutic gases employed in gas-propelled microneedles.

Gas	Representative gas source	Primary propulsion mechanism	Biological function	Representative applications	Advantages	Limitations	References
O_2_	CaO_2_, catalase, microalgae	Bubble generation/pressure	Relieves hypoxia, antibacterial	Wound healing, PDT	Dual propulsion + therapy	Storage stability, oxidative stress	[18,36,37]
H_2_	Mg, MgH_2_, NaBH_4_	Bubble generation	Antioxidant, anti-inflammatory	Wound healing, inflammatory diseases	Excellent biocompatibility	Low solubility, difficult sustained release	[38,39]
NO	NO donors	Local gas release	Antibacterial, angiogenesis	Infected wounds	Multifunctional signaling	Narrow therapeutic window, donor instability	[40,41]
CO	CO-releasing molecules	Gas release	Anti-inflammatory	Tissue repair	Potent signaling	Potential toxicity	[42,43]
CO_2_	Carbonate + acid	Bubble-driven convection	Mainly propulsion	Drug delivery	Strong propulsion	Local acidification	[21,44]
Others	H_2_S, NO_2_, SO_2_, etc.	Various	Gas signaling	Exploratory	Emerging potential	Limited evidence	[45,46]

### 3.1 Oxygen (O_2_)-Propelled microneedles

**Fig. 3:**
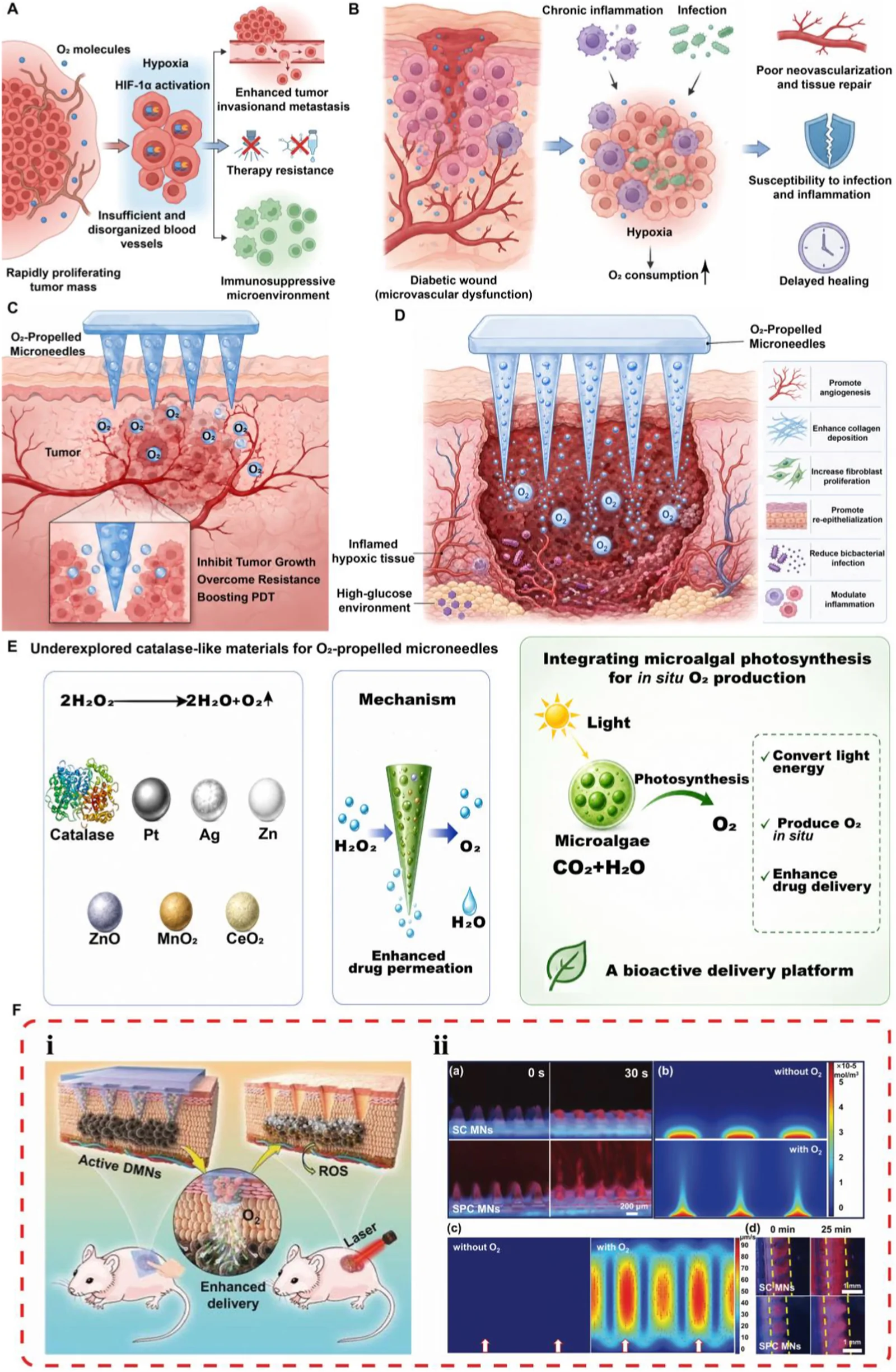
**O_2_-propelled microneedles alleviate hypoxia in tumors and diabetic wounds.** (A) Rapid tumor growth and abnormal vasculature cause hypoxia, leading to HIF-1α activation, tumor invasion, therapy resistance, and an immunosuppressive microenvironment. (B) Microvascular dysfunction and high glucose induce hypoxia, chronic inflammation, infection, and delayed healing. (C) O_2_-propelled microneedles deliver oxygen into tumors to overcome hypoxia and enhance therapeutic efficacy. (D) Microneedles supply O_2_ to promote angiogenesis, collagen deposition, fibroblast proliferation, reduce infection, and accelerate healing. (E) Catalase-like materials decompose H_2_O_2_ into O_2_ to enhance drug permeation. Integration of microalgal photosynthesis enables light-driven *in situ* O_2_ generation, forming a bioactive oxygen-producing delivery platform. F i) An illustration depicting Ce6-loaded active dissolving microneedles for improved photosensitizer delivery. The sodium carbonate reacts with dermal interstitial fluid to produce gaseous oxygen bubbles, acting as a pump to facilitate the penetration of the PS into deep tumor tissues. ii) Assessment of the in vitro payload release performance of Ce6-loaded active sodium carbonate microneedles and Ce6-loaded passive solid-core microneedles [18].

O_2_ supply deficiency, which results in hypoxia, is a prominent characteristic of various diseases, particularly in advanced solid tumors and diabetic wounds. In advanced solid tumors, the rapid proliferation of cancer cells often outpaces the formation of new blood vessels, leading to inadequate O_2_ delivery to the tumor tissue [47,48]. This hypoxic environment not only contributes to the aggressive behavior of the tumor but also makes it more resistant to conventional therapies [49]. Furthermore, hypoxia can promote tumor metastasis and create an immune-suppressive microenvironment, complicating treatment outcomes [50,51]. As illustrated in Fig. 3A, the rapidly expanding tumor mass experiences limited oxygen availability due to inadequate and disorganized blood vessel formation [52]. The resulting hypoxic stress stabilizes and activates the key transcription factor HIF-1α, which orchestrates a broad transcriptional program that drives tumor invasion and metastasis, therapeutic resistance, and immune suppression. Collectively, these effects facilitate tumor adaptation to hypoxia and ultimately accelerate malignant progression [53]. Similarly, in diabetic wounds, inadequate oxygenation hinders the healing process. Diabetes can impair blood flow and damage small blood vessels, leading to insufficient O_2_ in the affected areas. This deficiency slows down cellular processes crucial for tissue repair and regeneration, increasing the risk of infection and chronic ulcers [[53], [54], [55], [56]]. Chronic hyperglycemia induces vascular endothelial injury and microcirculatory dysfunction, leading to persistent reductions in blood perfusion and oxygen supply [57]. Meanwhile, sustained inflammation and recurrent infection further elevate local oxygen consumption, collectively establishing a chronic hypoxic microenvironment. Importantly, hyperglycemia directly suppresses the stability and transcriptional activity of HIF-1α, thereby impairing the activation of pro-angiogenic and tissue repair pathways, including VEGF expression [58]. Consequently, a vicious cycle of “hypoxia-impaired repair-aggravated hypoxia” is formed, ultimately contributing to delayed healing and the progression to chronic, non-healing wounds (Fig. 3B).

Therefore, delivering O_2_ is vital for achieving effective treatment of diseases. Restoring local O_2_ availability is essential for relieving hypoxia. O_2_ plays a critical role in regulating cellular signaling and the local microenvironment through hypoxia-responsive pathways, most notably the HIF-1α axis. HIF-1α axis regulates the expression of downstream factors such as VEGF and thereby influences angiogenesis, cellular adaptation, inflammation, and tissue repair. In tumor and wound microenvironments, restoring O_2_ levels can consequently reshape the behavior of tumor, endothelial, and immune cells by alleviating hypoxia-associated signaling dysregulation, providing a mechanistic basis for O_2_-based therapeutic intervention. To address hypoxia and improve drug penetration simultaneously, oxygen-generating microneedle systems have been developed. These systems combine the physical barrier-disrupting capability of microneedles with *in situ* O_2_ production, enabling therapeutic enhancement. Microneedle-mediated oxygen delivery physically penetrates the skin or superficial tumor barriers, enabling direct release of oxygen or oxygen-generating components into the tumor tissue [59]. This strategy bypasses the abnormal, disorganized, and inefficient tumor vasculature, thereby rapidly increasing intratumoral oxygen tension [60]. Elevated oxygen levels can reverse tumor hypoxia, suppress aberrant activation of hypoxia-related signaling pathways such as HIF-1α, and reduce malignant phenotypes including enhanced glycolytic dependence, pro-angiogenesis, invasion, metastasis, and immunosuppression [61]. Moreover, improved oxygenation enhances the cytotoxic efficacy of radiotherapy by promoting oxygen-dependent free radical formation, increases the sensitivity of tumors to chemotherapy and immunotherapy, and facilitates normalization of the tumor microenvironment (Fig. 3C). O_2_-propelled microneedles utilize a dissolvable microneedle array to penetrate the skin barrier and directly deliver oxygen into the hypoxic tissue of deep diabetic wounds, thereby fundamentally alleviating local hypoxia under hyperglycemic conditions and disrupting the vicious cycle in which oxygen deficiency suppresses angiogenesis and collagen synthesis [36,37]. Adequate oxygen supply not only promotes fibroblast proliferation and accelerates re-epithelialization, but also inhibits anaerobic bacterial infection and mitigates excessive inflammation. In addition, improved oxygenation enhances cellular responsiveness to growth factors, ultimately overcoming delayed healing and recurrent infection in diabetic wounds and enabling rapid and effective tissue repair (Fig. 3D).

Chemical oxygen-generating compounds such as calcium peroxide (CaO_2_) and sodium percarbonate can react with physiological fluids to produce O_2_ [18,62,63]:

$$2{\text{Na}}_{2}CO_{4}+2\;H_{2}O\to {2\text{Na}}_{2}CO_{3}^{+}O_{2}+{2H}_{2}O$$

$$2\text{Ca}O_{2}+2H_{2}O\to 2\text{Ca}{\left(\text{OH}\right)}_{2}^{+}O_{2}$$

For example, CaO_2_-loaded microneedle patches release nanoparticles into wound tissue, where hydrolysis generates O_2_ and hydrogen peroxide, providing both oxygen supplementation and antibacterial effects [62]. Beyond oxygen supply, gas generation creates localized pressure and microfluidic convection, enhancing drug diffusion into deeper tissues. In addition, gas-propelled dissolving microneedles incorporating sodium percarbonate have demonstrated enhanced transdermal drug migration via O_2_ bubble formation, which induces fluid flow and improves tissue penetration. Specifically, increasing the sodium percarbonate loading progressively enhanced O_2_ generation, with the formulation containing the highest sodium percarbonate loading producing 3.98 ± 0.08 mg of gaseous O_2_ per liter of patch in PBS [18]. The generated bubbles appeared within approximately 10 s after exposure to the aqueous environment, and the model photosensitizer chlorin e6 (Ce6) migrated approximately 1 mm within 30 s, whereas no obvious migration was observed within the same period in the control microneedles without the O_2_-generating component (Fig. 3F). This active propulsion mechanism significantly increases photosensitizer delivery and alleviates tumor hypoxia, thereby amplifying PDT efficacy [18]. However, the direct incorporation of catalase, as well as various metals and metal oxides with catalase-like activity-including platinum, silver, zinc, manganese dioxide, cerium dioxide, and zinc oxide-as applications of O_2_-propelled microneedles remains relatively underexplored [64]. These materials can convert H_2_O_2_ into water and O_2_, generating bubbles or concentration gradients that enhance drug permeation. In addition, their oxygen production is primarily governed by chemical reaction kinetics, making precise spatiotemporal regulation difficult and raising concerns regarding premature oxygen release, local pH fluctuations, and inorganic reaction byproducts. Compared with the chemically driven oxygen-generating systems, biological oxygen generators represent an emerging alternative with distinct characteristics. The biological oxygen generators based on photosynthetic microorganisms offer the potential for sustained and environmentally responsive oxygen production under appropriate light conditions, while generally exhibiting improved biocompatibility. Consequently, exploring novel strategies that combine microalgal photosynthesis for oxygen production with microneedle technology holds great promise. For instance, using microalgae like Chlorella, which can efficiently convert light energy into chemical energy, could enhance the oxygen generation process [65]. By integrating these microorganisms into the microneedle system, we could potentially create a bioactive delivery platform that not only facilitates drug delivery but also continuously produces oxygen *in situ* (Fig. 3E). Despite these encouraging outcomes, oxygen-propelled microneedles still face several challenges. Their therapeutic performance largely depends on the efficiency and controllability of oxygen generation within the confined microneedle matrix. Premature oxygen release during storage may reduce delivery efficiency, whereas excessive oxygen production could potentially induce oxidative stress in surrounding tissues. Therefore, the therapeutic benefit of oxygen delivery should be balanced against potential dose-dependent oxidative stress, highlighting the importance of precisely controlling oxygen generation and release within an appropriate therapeutic window. In addition, the oxygen diffusion distance remains limited in severely hypoxic or poorly vascularized tissues, and long-term in vivo safety and manufacturing stability require further investigation before clinical translation.

### 3.2 Hydrogen (H_2_)-Propelled microneedles

**Fig. 4:**
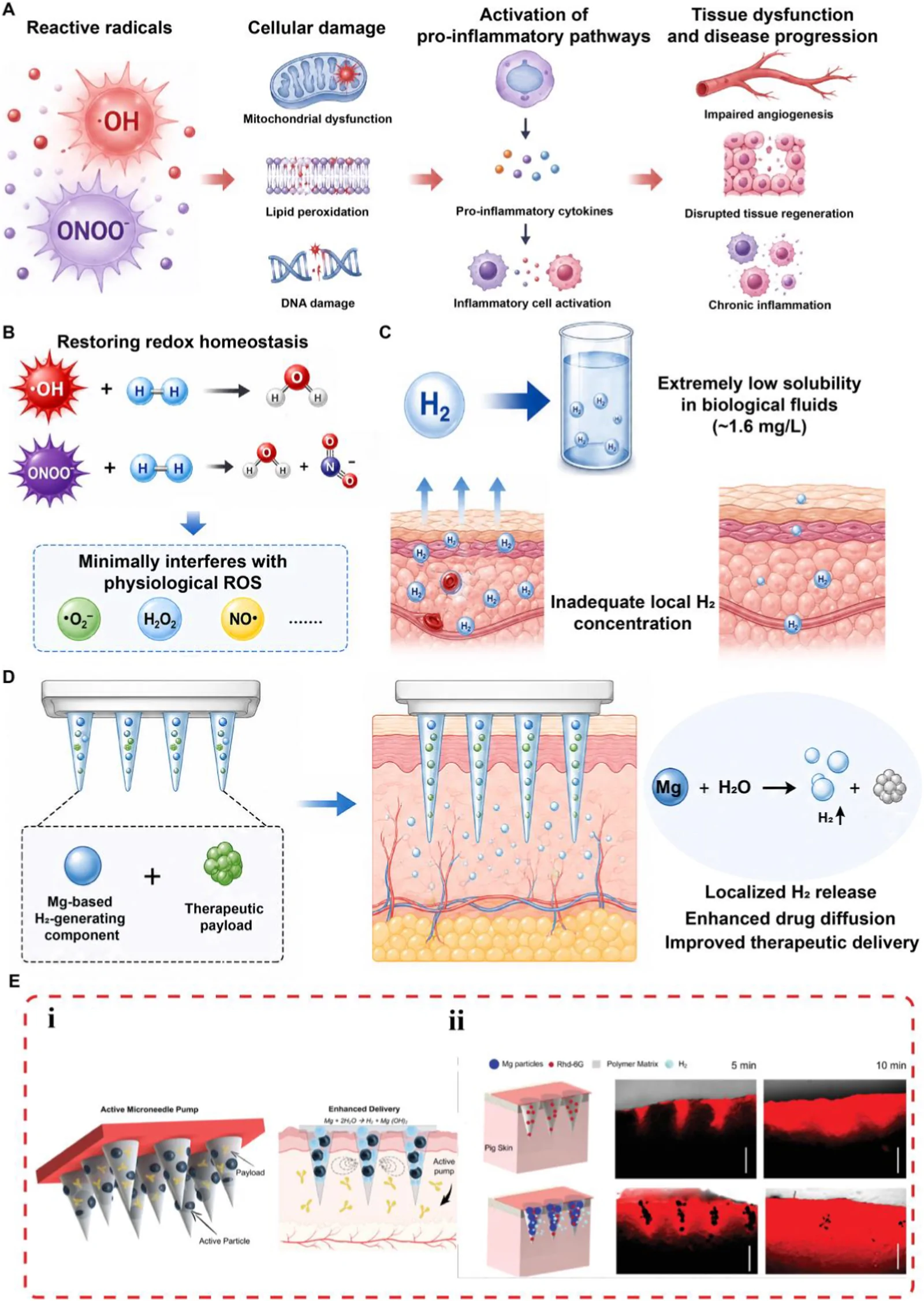
**Therapeutic rationale and mechanism of H_2_-propelled microneedle systems for enhanced localized delivery.** (A) Excessive reactive oxygen species, including hydroxyl radical and peroxynitrite, induce mitochondrial dysfunction, lipid peroxidation, and DNA damage, subsequently activating pro-inflammatory signaling pathways and promoting tissue dysfunction, impaired angiogenesis, disrupted tissue regeneration, and chronic inflammation. (B) Molecular H_2_ selectively scavenges highly reactive oxidants while minimally interfering with physiological ROS signaling. (C) Conventional hydrogen administration often results in insufficient local H_2_ concentration and limited therapeutic efficacy within diseased tissues. (D) Schematic illustration of the H_2_-propelled microneedle system. Upon insertion into tissue, the microneedles deliver Mg-based hydrogen-generating components and therapeutic payloads directly into the target site. E i) A schematic illustration of the active microneedle patch composition, highlighting the activation of built-in Mg particles as pumps upon contact with bodily fluids, which results in improved drug release. ii) A schematic illustrating the experimental setup for both passive and active microneedles penetrating porcine skin [69]. Copyright 2019, Wiley.

Under pathological conditions, overproduction of highly reactive radicals, particularly hydroxyl radicals and peroxynitrite, leads to mitochondrial dysfunction, lipid peroxidation, DNA damage, and activation of pro-inflammatory signaling pathways [66,67]. Persistent oxidative stress further amplifies inflammation, impairs angiogenesis, and disrupts tissue regeneration, thereby accelerating disease progression [68] (Fig. 4A).

H_2_ has been demonstrated to selectively scavenge strong oxidants such as hydroxyl radicals by converting them into H_2_O, while minimally interfering with physiological redox signaling [70]. Beyond its direct antioxidant activity, H_2_ can modulate redox-sensitive signaling pathways that regulate cellular inflammation, apoptosis, and stress responses. In particular, H_2_-mediated restoration of redox homeostasis has been associated with activation of the Nrf2/ARE antioxidant defense pathway and suppression of oxidative stress–responsive inflammatory signaling, thereby reducing the expression of pro-inflammatory mediators and protecting cells from oxidative injury. These signaling effects provide an important mechanistic link between H_2_-mediated ROS regulation and the subsequent modulation of cellular and immune responses within pathological microenvironments. In tumor tissues, modulation of redox homeostasis by H_2_ can suppress excessive glycolysis, reduce VEGF expression, alleviate immunosuppression, and in certain contexts disrupt mitochondrial respiration, ultimately inhibiting tumor progression [38,39]. In diabetic wounds and inflammatory dermatoses, H_2_ reduces oxidative damage, downregulates pro-inflammatory cytokines, promotes macrophage polarization toward the M2 phenotype, and restores tissue repair pathways [71] (Fig. 4B). However, similar to the insufficient O_2_ supply in hypoxic tissues, the therapeutic application of H_2_ is severely limited by its extremely low solubility in biological fluids (∼1.6 mg/L) [72]. Rapid diffusion and poor retention lead to inadequate local hydrogen concentration in lesion tissues, resulting in reduced bioavailability and compromised ROS-scavenging efficacy [73] (Fig. 4C). Therefore, strategies that enable sustained and localized H_2_ delivery are essential for maximizing therapeutic outcomes.

To address this limitation while simultaneously enhancing tissue penetration, H_2_-propelled microneedle systems have been developed. These systems integrate the physical barrier-disrupting capability of microneedles with *in situ* hydrogen generation (Fig. 4D). By penetrating the skin or superficial tumor barriers, microneedles directly deliver hydrogen-producing components into target tissues, allowing localized H_2_ release and rapid elevation of intralesional hydrogen concentration [74,75]. In addition to antioxidant effects, hydrogen bubble formation generates localized pressure gradients and microfluidic convection, enhancing drug diffusion into deeper tissues and improving therapeutic payload delivery. Miguel Angel Lopez-Ramirez et al. created a patch delivery system featuring a degradable polymeric microneedle array that contains a therapeutic payload, supplemented by active motor magnesium (Mg) microparticles [69]. The data indicated that the entrapped Mg particles significantly enhanced the delivery of the model dye Rh6G through both lateral and vertical routes, compared to the slower delivery of passive microneedles, which only began after 5 min. At the 10-min mark, passive microneedles achieved an average penetration of 111 ± 79 μm, while active microneedles reached 526 ± 71 μm (Fig. 4E).

Chemical hydrogen-generating materials have been widely incorporated into microneedle systems. Metals and metal hydrides such as magnesium (Mg), magnesium hydride (MgH_2_) [76], and sodium hydride (NaH) [24] react with physiological fluids to produce hydrogen gas:

$$\text{Mg}+2H_{2}O\to \text{Mg}(O{H)}_{2}^{+}H_{2}$$

$$2\text{NaH}\to 2\text{Na}+H_{2}$$

$$\text{Mg}H_{2}\to \text{Mg}+H_{2}$$

Upon contact with interstitial fluid, these reactions rapidly generate hydrogen bubbles due to the low solubility of H_2_ in water. The swift release of gas bubbles not only increases local hydrogen concentration but also provides mechanical propulsion, facilitating deeper penetration through dermal or tumor tissues [77]. Compared with passive microneedles, hydrogen-driven microneedles significantly enhance both lateral and vertical drug migration, thereby improving therapeutic efficacy [78]. Despite their advantages, metal-based hydrogen generation is constrained by material quantity, reaction controllability, and potential cytotoxicity of residual by-products [79]. To overcome these limitations, biologically driven hydrogen production strategies have been explored. Encapsulation of hydrogen-producing microorganisms, such as *Enterobacter aerogenes* or photosynthetic bacteria, within microneedles enables sustained gas generation while preserving microbial viability [19]. These bioengineered systems continuously produce hydrogen *in situ*, enhancing deep-tissue permeation and modulating oxidative stress in a more sustainable manner. Collectively, H_2_-propelled microneedles represent a multifunctional therapeutic platform that combines redox modulation, mechanical propulsion, and controlled drug delivery. By overcoming the intrinsic limitations of hydrogen's low solubility through *in situ* generation, this strategy enhances local antioxidant capacity, suppresses inflammation and tumor progression, and improves tissue regeneration. However, their clinical application remains constrained by the extremely low solubility and rapid diffusion of hydrogen, making sustained local retention difficult. Furthermore, precise regulation of hydrogen generation kinetics remains challenging. Insufficient hydrogen delivery may result in inadequate local exposure and fail to achieve the desired antioxidant and cytoprotective effects, whereas excessive or prolonged hydrogen accumulation may not necessarily confer additional therapeutic benefits and could potentially disturb local redox homeostasis or cause undesirable changes in the tissue microenvironment. Therefore, optimizing the dose and release kinetics of hydrogen is essential to maintain an effective therapeutic window, in which sufficient local hydrogen levels are achieved to exert therapeutic effects while avoiding unnecessary exposure and potential safety risks. Current studies are largely limited to preclinical animal models. Future optimization focusing on release controllability, biosafety, and integration with nanotechnology or synthetic biology may further expand the clinical translation of hydrogen-based microneedle systems.

### 3.3 Nitric oxide (NO)-Propelled microneedles

**Fig. 5:**
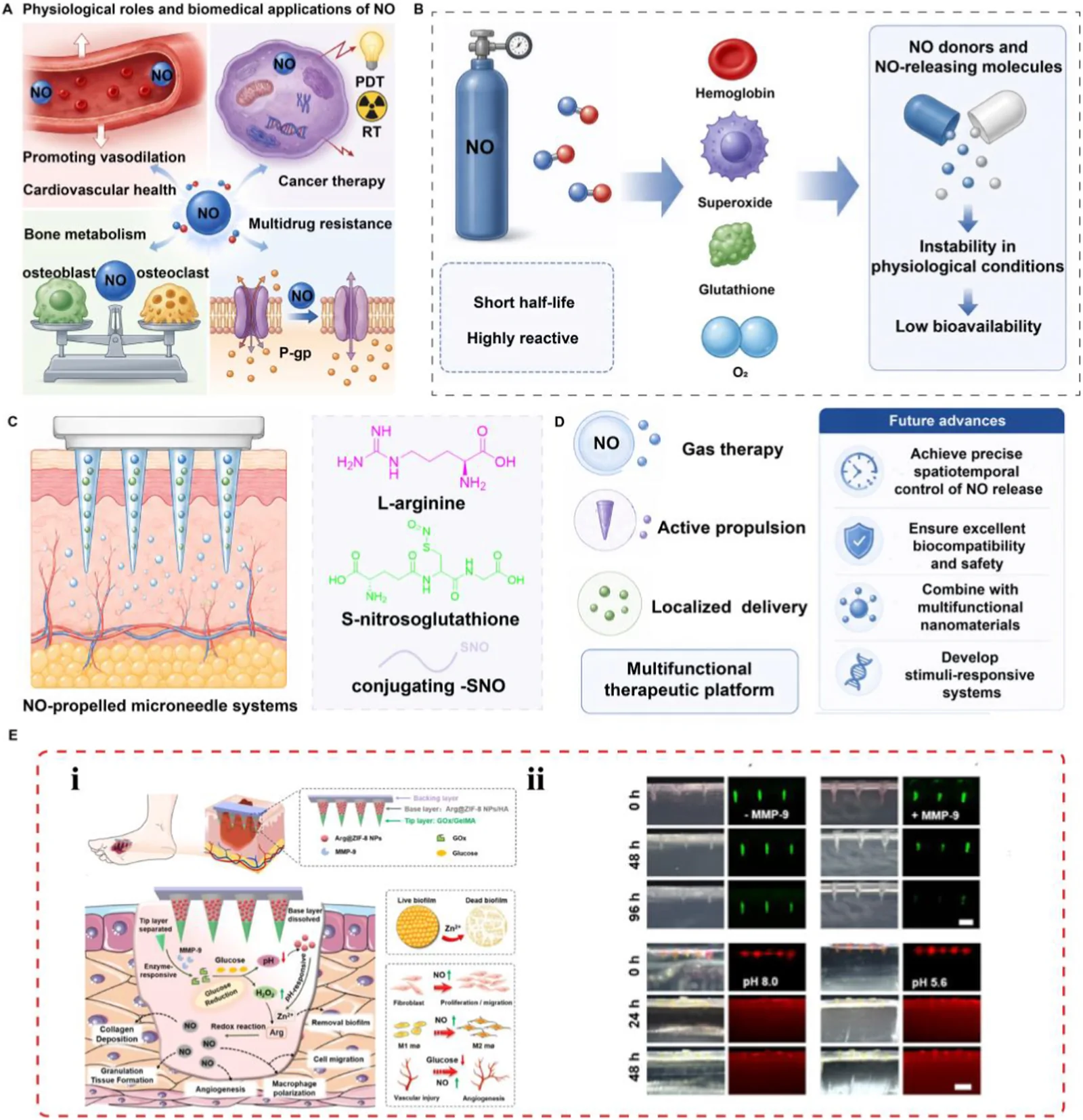
**Biomedical applications, limitations, and future perspectives of NO-propelled microneedle systems.** (A) NO serves as an important gaseous signaling molecule involved in multiple physiological and therapeutic processes. (B) Direct application of exogenous NO gas is limited by its short half-life and high reactivity toward biological substances. (C) Schematic illustration of NO-propelled microneedle systems. By integrating NO-generating components within microneedle platforms. (D) Future perspectives of NO-propelled microneedles as multifunctional therapeutic platforms integrating gas therapy, active propulsion, and localized drug delivery. E i) Schematic representation of the microenvironment-responsive bilayer G/AZ-MNs designed to enhance diabetic wound healing by depleting glucose and enabling the sustained release of NO. ii) Degradation of microneedles tips in agarose gels (simulated skin) with or without MMP-9 at different time points [88]. Copyright 2024, Elsevier.

NO is a pivotal gas transmitter involved in a wide array of physiological processes, including apoptosis, angiogenesis, and immune responses [41,80,81]. NO exerts these effects primarily through the soluble guanylate cyclase (sGC)/cyclic guanosine monophosphate (cGMP) signaling axis, while also regulating protein function through S-nitrosylation and interacting with redox-sensitive pathways. The NO–sGC–cGMP pathway is particularly important in vascular endothelial cells, where NO signaling promotes vasodilation and regulates angiogenic responses [82]. Its diverse roles in the body position it as a crucial player in several biomedical applications (Fig. 5A). In cardiovascular health, NO helps regulate blood flow and pressure by promoting vasodilation, thereby improving overall circulatory function [81,83]. In bone metabolism, NO contributes to the regulation of osteoblast and osteoclast activity, influencing bone density and health [84,85]. In the realm of cancer therapy, NO has garnered significant attention for its ability to induce cancer cell death through mechanisms such as the nitridation of mitochondrial components and DNA, which can lead to apoptosis. Beyond its direct cytotoxic effects on cancer cells, NO also plays a synergistic role in enhancing the efficacy of existing treatments like photodynamic therapy and radiotherapy. By improving oxygenation and sensitizing cells to these therapies, NO can potentially increase treatment effectiveness [40,41]. Moreover, NO addresses the challenge of multidrug resistance in cancer, often attributed to the overexpression of P-glycoprotein (P-gp) transporters that extrude therapeutic agents from cells, thus reducing their efficacy. By downregulating P-gp expression, NO offers a promising strategy for overcoming resistance and improving the success rate of chemotherapy [86]. However, the direct application of exogenous NO gas is hindered by its short half-life and susceptibility to various biological substances, including glutathione (GSH), hemoglobin, superoxide, and molecular O_2_ (Fig. 5B). Many NO donors and NO-releasing molecules exhibit extreme instability in physiological conditions, resulting in low bioavailability [87].

To address these limitations while simultaneously improving tissue penetration, NO-propelled microneedle systems have emerged as a promising therapeutic strategy (Fig. 5C). By combining the minimally invasive and barrier-disrupting properties of microneedles with controllable NO generation, these systems enable localized delivery and sustained release of NO directly within diseased tissues. Moreover, NO-driven gas propulsion can generate localized pressure gradients and microfluidic convection, thereby enhancing drug diffusion and deep-tissue penetration. Several advanced NO-releasing microneedle platforms have recently been developed. Incorporating the NO donor S-nitrosoglutathione [89], conjugating -SNO groups [90] and loading L-arginine molecules [88] enables controllable nitric oxide release. Leveraging the porous structure and mechanical strength of microneedles, this platform delivers NO accurately and deeply to target sites. Zeng et al. [88] developed the G/AZ-MNs, which feature a degradable tip layer that incorporates glucose oxidase (GOx) and a dissolvable base layer that encapsulates arginine-loaded nanoparticles (Arg@ZIF-8 NPs). Using a spatiotemporal responsive cascade strategy, the base layer of the patch rapidly dissolved after application to the wound, causing the tips of the microneedles to separate quickly. This separation then triggered a response to the overexpressed matrix metalloproteinase-9 in diabetic lesions, resulting in the responsive release of GOx. The released enzyme catalyzed the conversion of glucose into gluconic acid and H_2_O_2_, which not only reduced glucose levels in diabetic wounds but also initiated a cascade reaction between H_2_O_2_ and the arginine released from the nanoparticles, enabling a continuous production of NO for up to seven days (Fig. 5E). In addition to controlled release systems, NO-driven nanomotor-integrated microneedles have emerged as a novel approach for active drug transport [20,91]. Liu et al. [20] developed a detachable microneedle array to deliver exosomes modified by a NO nanomotor for the treatment of Achilles tendinopathy. A living small animal imaging system was employed to capture the fluorescence signals of these two substances. They observed the red fluorescence of MBA and the green fluorescence of EXO, indicating that EXO/MBA was effectively delivered into the hypodermis of the Achilles tendon region.

Currently, the major application areas of NO-propelled microneedles include tumor therapy, antimicrobial treatment, biofilm eradication, and ischemic tissue repair. L-arginine remains the most commonly used endogenous NO precursor because it can be converted into NO by nitric oxide synthase under oxidative stress conditions [20,92]. In addition, NO-storage materials such as copper-benzene-1,3,5-tricarboxylate frameworks have emerged as promising NO reservoirs for controllable release [93]. Nevertheless, several intrinsic limitations continue to hinder the clinical translation of NO-releasing Microneedles, including the difficulty of maintaining therapeutically relevant NO concentrations, avoiding excessive RNS generation, and ensuring adequate donor stability throughout storage and use [94]. Insufficient NO delivery may limit its beneficial effects on vasodilation, antimicrobial activity, and tissue repair, whereas excessive or sustained NO exposure may promote the formation of reactive nitrogen species (RNS), leading to oxidative/nitrosative stress and potential tissue damage. Thus, precise control of NO dosage and release kinetics is critical for maintaining an appropriate therapeutic window that maximizes its biological benefits while minimizing RNS-associated toxicity. Collectively, NO-propelled microneedles integrate gas therapy, active propulsion, and localized drug delivery into a multifunctional therapeutic platform. Future advances focusing on controllable NO release, biosafety, and synergistic integration with nanotechnology and responsive biomaterials may further accelerate their clinical translation (Fig. 5D).

### 3.4 Carbon monoxide (CO)-Propelled microneedles

**Fig. 6:**
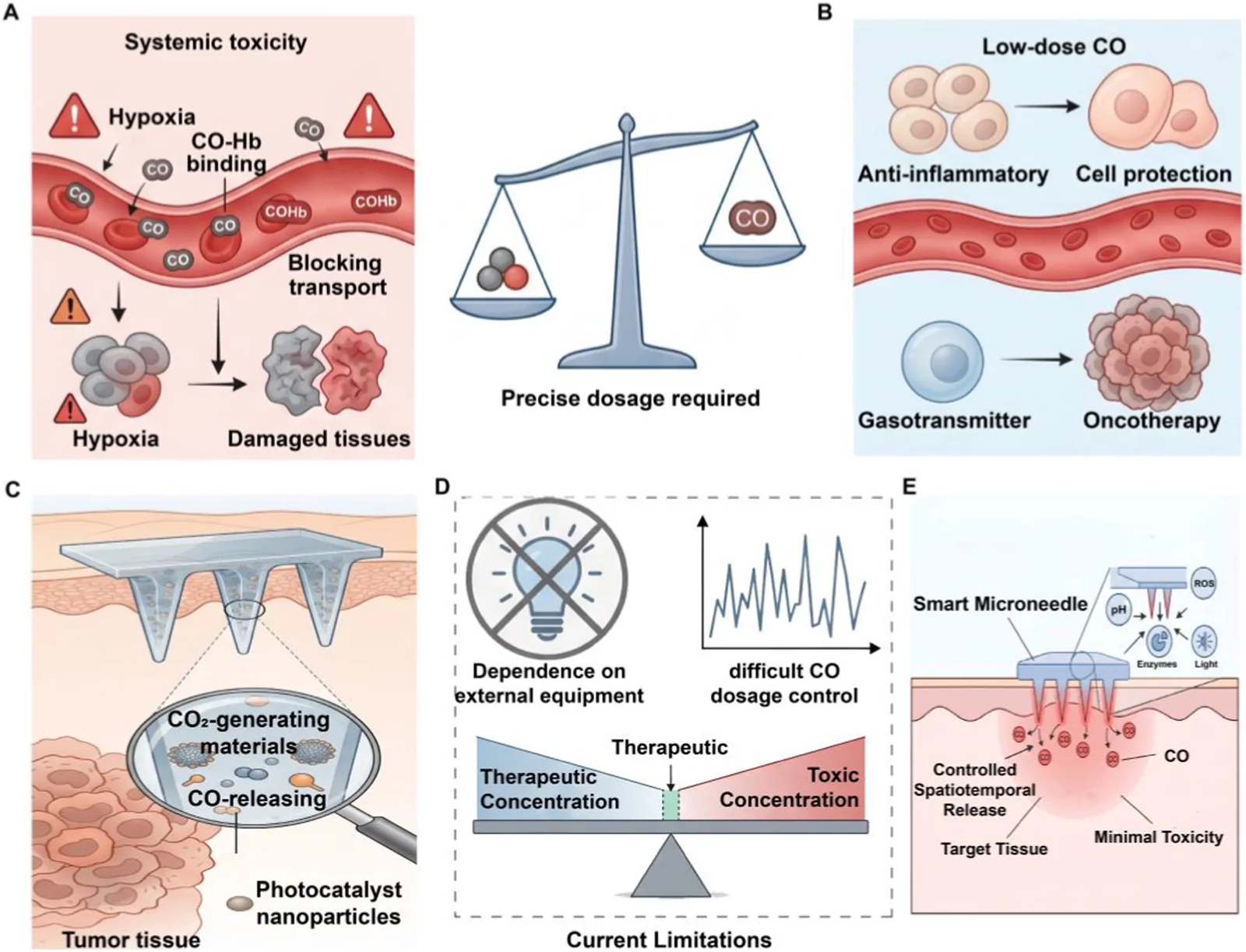
**Schematic illustration of the dual biological effects, current limitations, and future development strategies of CO-propelled microneedles.** (A) Excessive CO binds to hemoglobin (CO-Hb), blocking oxygen transport and inducing systemic toxicity, hypoxia, and tissue damage. (B) CO functions as a gasotransmitter with anti-inflammatory, cytoprotective, and tumor therapeutic effects. (C) Cascade-activated CO microneedle systems utilize CO_2_-generating materials and photocatalyst nanoparticles to achieve localized CO release within diseased tissues. (D) Current limitations of CO-related microneedles. (E) Future smart microneedle platforms with stimulus-responsive properties.

CO is widely recognized as a toxic gas that can lead to poisoning by interfering with hemoglobin's ability to transport O_2_ when present in excessive amounts (Fig. 6A). However, recent studies have uncovered that low concentrations of CO can function as a signaling molecule, playing a regulatory role in various physiological processes within the nervous, cardiovascular, and immune systems. Specifically, CO can modulate the sGC/cGMP axis and activate cytoprotective pathways such as the Nrf2/antioxidant response element (ARE) pathway, thereby influencing oxidative stress, inflammation, apoptosis, and vascular responses. In immune and inflammatory microenvironments, these signaling effects can modulate the activity of immune and parenchymal cells, contributing to the resolution of excessive inflammation and protection against cellular injury. This emerging understanding has prompted researchers to explore the therapeutic potential of CO in treating a range of medical conditions, including inflammation [95], gastrointestinal disorders [96], arteriosclerosis [97], strokes [98], and even cancer [99] (Fig. 6B).

The therapeutic effects of CO are highly dependent on its local concentration and exposure duration, with insufficient CO exposure potentially limiting its cytoprotective and anti-inflammatory effects, whereas excessive or prolonged exposure may lead to CO accumulation, impaired oxygen transport, and tissue hypoxia. Therefore, precise control of CO concentration is critical for maintaining an appropriate therapeutic window that maximizes its therapeutic benefits while minimizing toxicity. To harness the beneficial effects of CO while mitigating its toxic properties, there is an urgent need to develop controlled CO administration strategies. These strategies aim to ensure the safe and effective delivery of CO in a manner that allows for precise and on-demand release, optimizing its therapeutic effects while minimizing risks associated with its toxicity. One promising approach involves the use of CO-releasing molecules, which can release CO in a controlled manner under specific physiological conditions. This targeted release can facilitate the therapeutic use of CO in treating diseases by providing localized delivery, thereby enhancing its efficacy while reducing systemic exposure [100]. Furthermore, ongoing research is focused on understanding the signaling pathways through which CO exerts its effects. For example, studies have demonstrated that CO can activate specific intracellular signaling cascades, leading to anti-inflammatory responses and promoting cell survival under stress conditions [101]. This dual action of CO-both as a toxic gas at high concentrations and as a therapeutic agent at lower concentrations-highlights the importance of careful dosing and administration protocols.

To further improve localized administration and therapeutic precision, microneedle-based delivery systems have been explored for CO-associated therapy. By combining minimally invasive microneedle technology with controlled gas generation, these systems enhance transdermal delivery while allowing localized therapeutic action. However, unlike O_2_, H_2_, or NO, CO itself is not an ideal propelling gas because effective gas-propelled permeation generally requires relatively high gas production, which raises significant safety concerns due to CO toxicity. Consequently, current CO-related microneedle systems often rely on indirect cascade strategies in which other gases, particularly CO_2_, are first generated to enhance tissue permeation, followed by subsequent catalytic conversion into therapeutic CO [42,43] (Fig. 6C).

Although promising, current CO-propelled microneedle systems still face several limitations, including process complexity, dependence on external photocatalytic equipment, and difficulties in achieving precise dosage control. Moreover, because the therapeutic window of CO is extremely narrow, excessive gas production may result in systemic toxicity and impaired oxygen transport (Fig. 6D). Therefore, future development of CO-associated microneedle systems should focus on improving controllability, biosafety, and stimulus responsiveness to enable precise spatiotemporal CO release (Fig. 6E).

### 3.5 Carbon dioxide (CO_2_)-Propelled microneedles

**Fig. 7:**
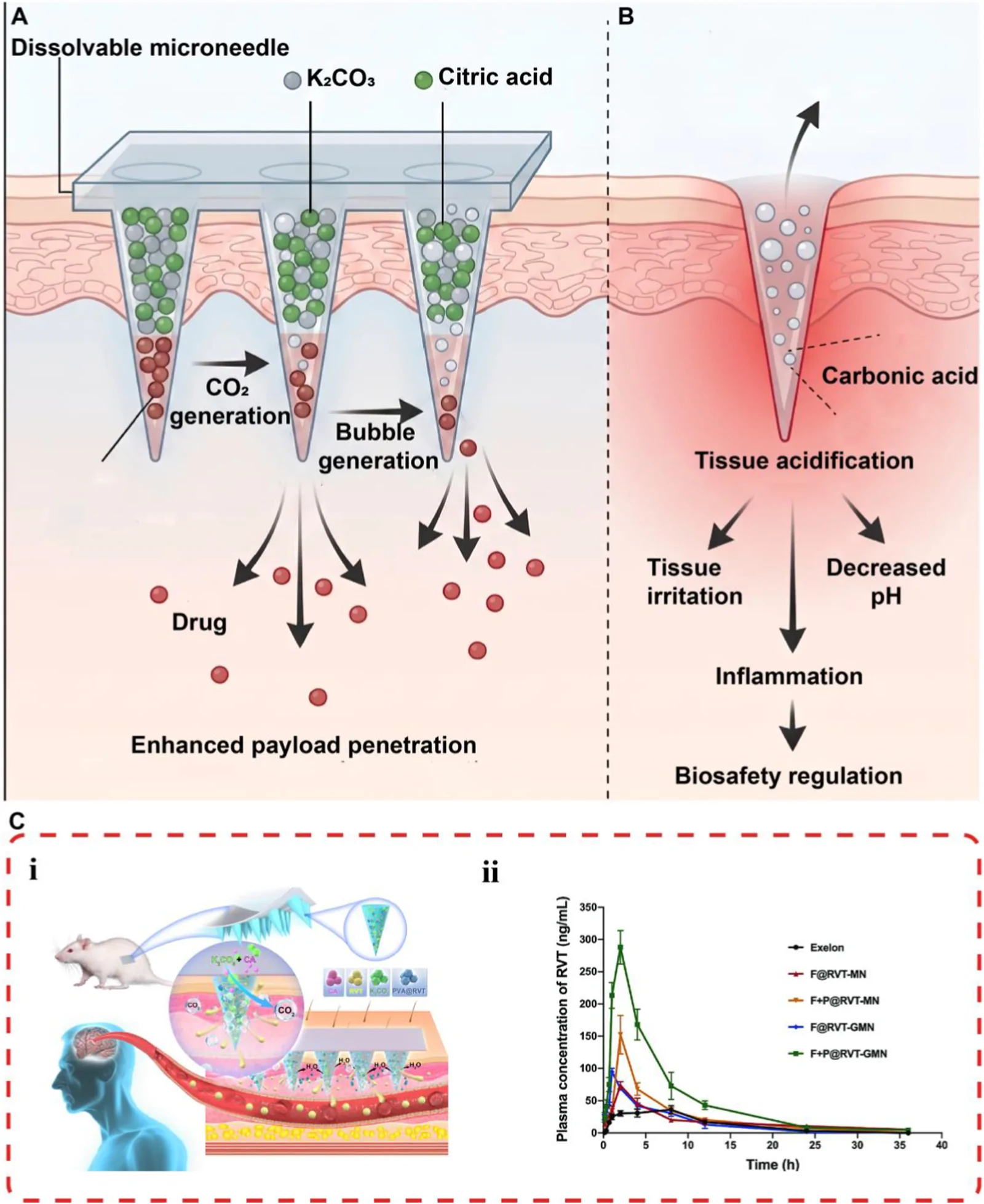
**Schematic illustration of CO_2_-propelled dissolvable microneedles for enhanced transdermal drug delivery and their potential limitations.** (A) Dissolvable microneedles incorporating potassium carbonate and citric acid generate CO_2_ bubbles upon contact with interstitial fluid. (B) Excessive CO_2_ generation may form carbonic acid in biological fluids, leading to local tissue acidification, decreased pH, irritation, and inflammatory responses. C. i) Schematic representation of guava-inspired gas-propelled microneedles for the early intervention and sustained treatment of Alzheimer's disease. ii) Pharmacokinetic profiles of Exelon patch and different microneedles [21]. Copyright 2024, Elsevier.

CO_2_ itself does not possess specific therapeutic functionality. However, when combined with microneedles, can significantly enhance drug penetration and increase the effective payload of medications [102]. To exploit this mechanism, gas-generating components are commonly incorporated into dissolvable or biodegradable microneedles. Upon contact with interstitial fluid, these materials rapidly generate CO_2_ bubbles, which provide mechanical propulsion that accelerates payload diffusion into deeper skin layers (Fig. 7A). The rapid expansion of CO_2_ bubbles within the confined microenvironment generates localized pressure gradients, which produce convective fluid movement and facilitate active transport of therapeutic agents beyond passive diffusion. From a physical perspective, the pressure generated by CO_2_ expansion can be approximately described by the ideal gas law (PV=NRT), indicating that rapid gas generation within a confined volume can result in a transient increase in local pressure. Meanwhile, the pressure difference across the curved gas–liquid interface can be described by the Young–Laplace relationship ($\Delta P=\gamma \left(\frac{1}{R1}+\frac{1}{R2}\right)$), highlighting the contribution of bubble size and interfacial tension to local pressure generation. The resulting pressure gradients can drive interstitial fluid movement within the tissue, which can be theoretically described by Darcy's law ($Q=-\frac{kA}{\mu }\frac{\Delta P}{L}$). In this process, gas-induced convection provides an additional transport component to molecular diffusion, thereby enhancing the distribution of therapeutic agents into deeper tissues. Therefore, CO_2_ propulsion can be regarded as a coupled gas–fluid transport process, in which gas generation and bubble expansion generate localized pressure gradients that are subsequently converted into fluid convection and enhanced payload transport [19]. The magnitude and duration of this propulsion effect depend on factors such as the rate and amount of CO_2_ generation, bubble size and distribution, the degree of spatial confinement, and the permeability of the surrounding tissue. Rapid gas generation may produce stronger transient propulsion, whereas controlled and sustained gas production may favor prolonged drug transport. However, excessive gas generation or pressure accumulation may compromise tissue safety. Thus, rational control of CO_2_ generation and bubble expansion is essential for balancing propulsion efficiency, drug penetration, and biosafety [19]. Using potassium carbonate and citric acid as a gas-propelled driving system is the most prevalent strategy to further improve the transdermal delivery efficiency of drugs [21,44]. For example, zhang et al. employed K_2_CO_3_ and citric acid as pneumatic engines to introduce microneedles, enhancing the penetration of free rivastigmine into deeper skin layers for early intervention in Alzheimer's disease [21]. Compared to microneedles loaded with free rivastigmine, the bionic microneedles powered by the dual-engine approach exhibited increases in drug loading capacity, cumulative transdermal permeability, and standardized bioavailability of approximately 40%, 22%, and 49%, respectively (Fig. 7C).

Despite these advantages, the use of CO_2_ in gas-propelled microneedle systems is not without limitations. When dissolved in biological fluids, CO_2_ forms carbonic acid, which may reduce local pH and induce tissue acidification. Excessive acidification can potentially trigger irritation, inflammatory responses, or discomfort at the administration site [103]. Therefore, careful regulation of CO_2_ generation rate and local concentration is essential to ensure biosafety and maintain tissue compatibility (Fig. 7B).

### 3.6 Other gas-propelled microneedles

Although oxygen, hydrogen, nitric oxide, carbon monoxide, and carbon dioxide currently dominate the development of gas-propelled microneedle systems, several other gaseous mediators have also attracted increasing attention because of their unique biological activities and potential compatibility with microneedle-based delivery. Although studies remain limited, these gases represent emerging candidates for next-generation gas-propelled microneedle platforms and are discussed here to highlight future research directions. Hydrogen sulfide (H_2_S) has various applications in the medical field, including anti-inflammatory effects, cardiovascular protection, neuroprotection, and antioxidant properties [104,105]. It plays a role in regulating immune responses, dilating blood vessels, and lowering blood pressure, indicating its potential therapeutic effects for conditions such as heart disease and hypertension [105]. Additionally, H_2_S promotes cell growth and repair, which may be beneficial in tissue regeneration and wound healing [106]. Recent studies have also highlighted the importance of H_2_S in the tumor microenvironment and its protective effects in respiratory diseases, underscoring its potential value as a biological signaling molecule in medical research and clinical treatment [45,46]. Beyond these established applications, ongoing research is exploring the role of H_2_S in various other health contexts. For instance, its modulation of mitochondrial function and its influence on metabolic processes present exciting opportunities for developing new therapeutic strategies [107]. The gas's involvement in neuroprotection suggests potential applications in neurodegenerative diseases, while its anti-inflammatory properties might be harnessed to address chronic inflammatory conditions. Furthermore, as a signaling molecule, H_2_S interacts with other gases, such as NO and CO, which could lead to synergistic effects in various biological processes. This intricate interplay opens avenues for innovative treatments, especially in conditions where inflammation and oxidative stress play pivotal roles [108]. Hu et al. developed a degradable microneedle with exceptional mechanical properties by optimizing the ratios of poly(vinyl alcohol), poly(vinyl pyrrolidone), and sodium hyaluronate. This microneedle was designed to deliver glucose oxidase (GOx) for tumor starvation and diallyl trisulfide for gas therapy. Controlled release of GOx and H_2_S was facilitated through ultrasound or near-infrared laser irradiation [35]. This study demonstrates the feasibility of integrating H_2_S donors into microneedle platforms for localized gas delivery. Compared with oxygen- or hydrogen-based systems, H_2_S-propelled microneedles remain at an early stage of development. Nevertheless, their unique gasotransmitter properties and controllable local delivery enabled by microneedles suggest considerable potential for future applications in wound repair, inflammatory diseases, and tumor therapy.

Other specific gases have also shown potential effectiveness, although they are still in the preliminary stage, with only a limited number of reports available. Nitrogen dioxide (NO_2_) is primarily used in respiratory therapy, where it can dilate the airways and improve symptoms of acute respiratory diseases. Nitrous oxide (N_2_O) is commonly used for anesthesia and analgesia, particularly in dental procedures, as it effectively alleviates patient anxiety and pain [109]. Sulfur dioxide (SO_2_) has shown anti-inflammatory effects in certain studies, but it should be used cautiously to prevent respiratory irritation [110]. To date, the integration of these gaseous mediators into microneedle systems remains largely unexplored. Nevertheless, their diverse biological activities suggest promising opportunities for future gas-propelled microneedle platforms. Precise regulation of gas generation is particularly important, as insufficient gas production may fail to achieve the desired therapeutic effects, whereas excessive generation may increase the risk of concentration-dependent toxicity and adverse tissue responses. Further investigations are required to establish appropriate gas donors, controllable release strategies, and disease-specific therapeutic applications before clinical translation can be realized.

## 4 Design strategies for gas-propelled microneedles

### 4.1 Microneedle materials

When manufacturing microneedles, the ability to insert effectively is arguably one of the most important factors. However, other considerations must also be taken into account, including cost, biocompatibility, and scalability for mass production. Therefore, the selection of materials should be the result of a comprehensive and multifaceted evaluation.

Currently, a variety of materials have been utilized for microneedle manufacturing based on different application requirements, including metals, silicon, ceramics, and polymers [111,112]. Polymers are widely regarded as the most popular materials for microneedle manufacturing due to their versatility, biodegradability, biocompatibility, strength ratios, low toxicity, and cost-effectiveness [7,113]. Because of their high biocompatibility, polymers are primarily used to create dissolvable and hydrogel-forming microneedles, alleviating concerns about potential residual microneedle materials left in the skin after insertion [33]. Typically, polymers exhibit greater toughness than ceramics or glass, although their strength is lower than that of metals, silicon, ceramics, and glass. Fortunately, various polymer blends can be utilized to achieve the mechanical properties required for specific clinical applications [114]. Microneedles can be constructed from various polymers, and categorized into biodegradable options like polylactic acid (PLA) and polyvinyl alcohol (PVA), which are suitable for short-term drug release, and hydrogels like hyaluronic acid (HA), chitosan, polyethylene, and polyethylene glycol diacrylate (PEGDA) that offer excellent water absorption and biocompatibility for controlled release applications [115]. When employing specific active agents such as *Clostridium perfringens* and Lactobacillus as gas sources, the utilization of biodegradable materials like PLA, HA, and chitosan for microneedle fabrication is preferable to ensure biological activity and facilitate sustained gas production [19]. Furthermore, when utilizing drug molecules or metallic substances as gas sources, materials such as PLA and PVA are more appropriate. These materials guarantee that the drug molecules or metallic components can rapidly interact with bodily fluids, resulting in a substantial release of gas to enhance drug permeation [24,69]. Non-biodegradable polymers, such as polyurethane and polystyrene, offer mechanical strength suitable for long-term applications, while conductive polymers like polypyrrole and polyaniline facilitate electrically stimulated drug delivery and biosensing [116]. In situations where prolonged stimulation or high mechanical strength is needed for gas production, the aforementioned materials are a more advantageous choice.

For gas-propelled microneedles, material selection should additionally consider the interaction between the microneedle matrix and the gas-generating components. The mechanical strength of the matrix must be sufficient to maintain structural integrity during insertion and gas generation, while its swelling, dissolution, or degradation behavior can determine the accessibility of body fluids to the gas source and thereby influence gas-generation kinetics. Accordingly, materials with rapid dissolution or high water permeability may favor rapid gas generation and transient propulsion, whereas slowly degrading or structurally robust matrices may be advantageous when prolonged gas production and sustained propulsion are desired. Therefore, the material should be selected not only according to its ability to withstand skin insertion but also according to its capacity to regulate the timing, magnitude, and duration of gas generation.

Hence, the most suitable gas-propelled microneedle materials are typically polymer-based substrates, such as PVA or PLA. These materials offer excellent biocompatibility and appropriate mechanical strength, effectively supporting the microneedle structure. Additionally, the tunability and ease of processing of these polymers allow for the design of various microneedle shapes and sizes tailored to specific application needs, thereby enhancing drug penetration into target tissues. Additionally, the tunability and ease of processing of these polymers allow for the design of various microneedle shapes and sizes tailored to specific application needs, thereby enhancing drug penetration into target tissues. Furthermore, previous studies have systematically investigated and summarized the effects of different polymeric materials and formulations on the mechanical strength of microneedles, providing useful guidance for material selection and structural optimization in the design of gas-propelled microneedle systems [117,118].

Beyond these conventional materials, a growing body of research has focused on advanced synthetic polymers and functional composite materials that can impart additional responsive or programmable functions to microneedles [119]. By incorporating stimuli-responsive moieties, conductive components, dynamic crosslinking networks, or nanomaterials, these emerging materials can respond to external or endogenous stimuli such as light [120], temperature [121], pH [122], electric fields [123], and biochemical signals, enabling more precise control over drug release and therapeutic activity. For example, temperature-responsive polymers such as poly(N-isopropylacrylamide) (PNIPAM) [121] and conductive polymers such as polypyrrole (PPy) [123] have been explored to construct stimuli-responsive microneedles, while nanocomposite systems incorporating metallic or carbon-based nanomaterials can further integrate photothermal, electrical, or catalytic functions. Such multifunctional materials provide new opportunities for integrating sensing, stimulus-responsive release, and localized therapy within a single microneedle platform, and may be particularly attractive for future gas-propelled microneedles requiring spatiotemporally controlled gas generation and therapeutic delivery.

### 4.2 Microneedle geometry

The skin is fundamentally elastic, and thus the microneedles must be designed to overcome this elasticity to achieve adequate penetration. Careful consideration must be given to the geometry of the microneedles array, including the spacing between needles, the length, and thickness of needles, the shape of the needles, as well as the diameters of the needle tips and bases, to successfully penetrate the skin with minimal pain and enhancing drug permeation. The types of microneedles significantly influence drug permeation based on their shapes. Sharp-tipped microneedles, due to their pointed design, penetrate the skin more easily, requiring less force and enabling faster permeation. In contrast, blunt-tipped microneedles may result in higher pain levels and slower permeation. Conical microneedles are ideal for rapid drug delivery, while rounded microneedles are better suited for uniform release. Computed tomography results indicate that increasing the number of vertices (hexagonal > square > triangular) can enhance the mechanical properties of the needles. However, a higher vertex count also decreases the needles' ability to penetrate the skin [124]. Long microneedles can reach deeper layers of skin but may cause discomfort, whereas short microneedles are more suitable for surface-level applications and tend to be less painful. While fine microneedles possess strong penetration capabilities, they may have slower release rates; on the other hand, thicker microneedles penetrate more effectively but can lead to greater discomfort [125]. For example, insertion forces of approximately 0.1–3 N have been reported across different microneedle geometries, with the insertion force increasing linearly with the interfacial area of the needle tip, highlighting the importance of geometric parameters in penetration performance [126]. In terms of needle density, a phenomenon known as the "bed-of-nails" effect can occur. While increasing the number of needles per square centimeter on a single baseplate seems beneficial for enhancing the drug delivery capacity of microneedle arrays, applying the same force to a higher-density microneedle array can lead to a lower force exerted on each needle. This reduced force may hinder needle insertion and consequently decrease drug delivery effectiveness. This phenomenon has been well-documented in the literature [127,128]. Moreover, the effects of geometric parameters, including needle length, tip diameter, aspect ratio, and spacing, on microneedle mechanical strength and insertion performance have been systematically reviewed elsewhere, providing practical design guidance for optimizing the balance between mechanical robustness, penetration efficiency, and patient comfort [129]. Optimizing microneedle design is essential for balancing drug permeation rates and pain levels.

The optimal gas-propelled microneedle morphology is characterized by a conical or tapered configuration. This design effectively penetrates the epidermal layer, thereby minimizing discomfort during insertion while maximizing surface area to enhance both gas release and drug permeation. The dimensions of the microneedles-specifically their length and diameter-should be meticulously optimized to ensure structural integrity during skin penetration and to promote efficient drug delivery alongside gas generation. In addition, for gas-propelled systems, needle density should also be considered together with the amount and distribution of the gas source. A high-density array may increase the overall drug-loading capacity but reduce the mechanical force delivered to each individual needle during insertion. Similarly, an uneven distribution of gas-generating components may result in heterogeneous gas generation and consequently nonuniform drug penetration. Therefore, the spatial arrangement of the gas source within the microneedle array represents an additional design parameter for achieving reproducible and spatially controlled drug delivery.

### 4.3 Selection of gases

In gas-propelled microneedle delivery systems, the relationship between the gas and the delivery system is inherently complementary. Therefore, gas selection should first consider its capacity to generate effective and controllable propulsion, followed by its potential intrinsic therapeutic activity when applicable. From the perspective of propulsion, key parameters include the amount and rate of gas generation, bubble formation and expansion, the duration of gas production, and the ability to convert gas expansion into localized pressure or mechanical forces. A gas source capable of rapid gas generation may produce a strong but transient propulsion effect, whereas a slowly activated gas source may provide more sustained propulsion. Accordingly, the appropriate gas should be selected according to the desired penetration depth, drug type, duration of delivery, and required degree of spatiotemporal control. From a therapeutic perspective, we should select the appropriate gas based on the specific indications. For example, the introduction of O_2_ exhibits high biocompatibility and can significantly enhance the therapeutic effects of treatments typically associated with O_2_, such as photodynamic therapy, radiotherapy, and sonodynamic therapy. Additionally, O_2_ can improve the hypoxic environment of wounds, thereby promoting the healing process [130,131]. Due to its ability to react with free radicals, H_2_ is particularly suitable for the treatment of diseases related to oxidative stress [132]. By selectively neutralizing harmful reactive oxygen species, H_2_ can help mitigate cellular damage and inflammation. This makes it a promising therapeutic option for various conditions, including neurodegenerative disorders, cardiovascular diseases, and metabolic syndromes [133]. Due to its vasodilatory and anti-inflammatory properties, NO is particularly well-suited for the treatment of cardiovascular diseases [134]. CO, despite its well-known toxicity, can promote bronchodilation and reduce inflammation at low doses, making it potentially useful for treating respiratory diseases [42,96,100]. CO_2_ molecules themselves do not possess specific therapeutic functions for treating diseases. However, when used in conjunction with microneedles, they typically serve as a propellant to enhance drug penetration.

On the other hand, from the perspective of gas-assisted permeation, selecting the appropriate gas source is crucial. For example, using magnesium-based materials can generate a significant amount of gas bubbles in a short time, greatly enhancing drug permeation. However, this method may lead to metal residues, and the effect is not long-lasting [135]. Moreover, hazardous gases like CO cannot promote drug permeation when produced in insufficient quantities. However, excessive production can cause irreversible damage to the body [136]. Therefore, maintaining a balance between the two is crucial. Utilizing nanomotors or microorganisms as gas sources is a highly promising strategy [19,137]. This approach not only leverages the inherent chemotaxis of the carriers to enhance the targeted accumulation of drugs in disease sites but also enables sustained gas production, leading to longer-lasting effects.

In the selection of gases for gas-propelled microneedles, it is imperative to choose the appropriate gas based on specific criteria rather than employing a one-size-fits-all methodology. Furthermore, considerations regarding gas sources should encompass factors such as biodegradability and biocompatibility. The utilization of biodegradable gas sources can significantly mitigate environmental impact and diminish the risk of accumulation within biological systems. Ensuring biocompatibility is essential to prevent adverse immune responses or irritation at the site of application.

### 4.4 Vehicles in microneedles

In the context of microneedle technology, many modern designs incorporate carriers such as nanoparticles, cells, and even bacteria, enhancing the therapeutic capabilities of microneedles beyond simple delivery systems.

When combined with microneedles, nanoparticles typically serve two primary roles: as therapeutic agents or as drug carriers. In the first case, materials with photothermal properties—such as gold, silver, and zinc oxide-along with multifunctional enzyme materials like cerium oxide and polydopamine-are widely employed [138,139]. In their role as drug carriers, various nanoparticles, including poly(D, L-lactide-co-glycolide) nanoparticles, poly(lactic-co-glycolic acid) nanoparticles, and calcium phosphate particles, are used in conjunction with microneedles. Nanoparticles are typically combined with gas donor molecules through loading or doping methods to achieve deep drug delivery [84,140]. These nanoparticles are often integrated into dissolvable microneedles through techniques such as copolymerization, solvent evaporation, or coating the surfaces of solid microneedles [64].

Therapy using active biomolecules is a well-established and promising approach. Mesenchymal stem cells (MSCs), induced pluripotent stem cells (iPSCs), adipose-derived stem cells (ADSCs), and endothelial progenitor cells (EPCs) are all viable options due to their versatility [141,142]. In addition, lactic acid bacteria, *Escherichia coli*, and unicellular algae play significant roles in the medical field due to their unique biological properties [65]. Currently, gas-producing microalgae and gas-producing bacteria have been applied in the field of biomedicine [19,143]. These active biomolecules can be integrated with microneedles in three main ways: 1) embedded within the microneedles, 2) seeded onto the microneedle surface, or 3) delivered directly through hollow microneedles. However, encapsulating biomolecules within microneedles presents challenges, as the fabrication processes often require harsh conditions—such as high temperatures, chemical solvents, ultraviolet light, or vacuum environments. Moreover, issues related to contamination, long-term storage, and maintaining viability pose significant hurdles in this approach [144].

For living gas-producing vehicles, the balance between viability and gas-generation capacity is particularly important. The microenvironment within the microneedle, including water availability, nutrient accessibility, and matrix degradation, may directly influence the activity of the incorporated microorganisms or cells and consequently determine the temporal profile of gas generation. Therefore, vehicle selection should be coordinated with microneedle material and gas-source design to achieve the desired duration and magnitude of propulsion while maintaining biological activity and safety.

In conclusion, the selection of carriers characterized by biocompatibility, stability, and ease of processing is paramount for the effective application of gas-propelled microneedles in drug delivery systems. These vehicles not only proficiently safeguard and release therapeutic agents but also ensure their bioactivity and efficacy within the physiological environment. Furthermore, versatile bioactive molecules such as mesenchymal stem cells and microalgae can offer a diverse array of biological functions, thereby facilitating the integration of gas therapies and broaden potential applications.

**Fig. 8:**
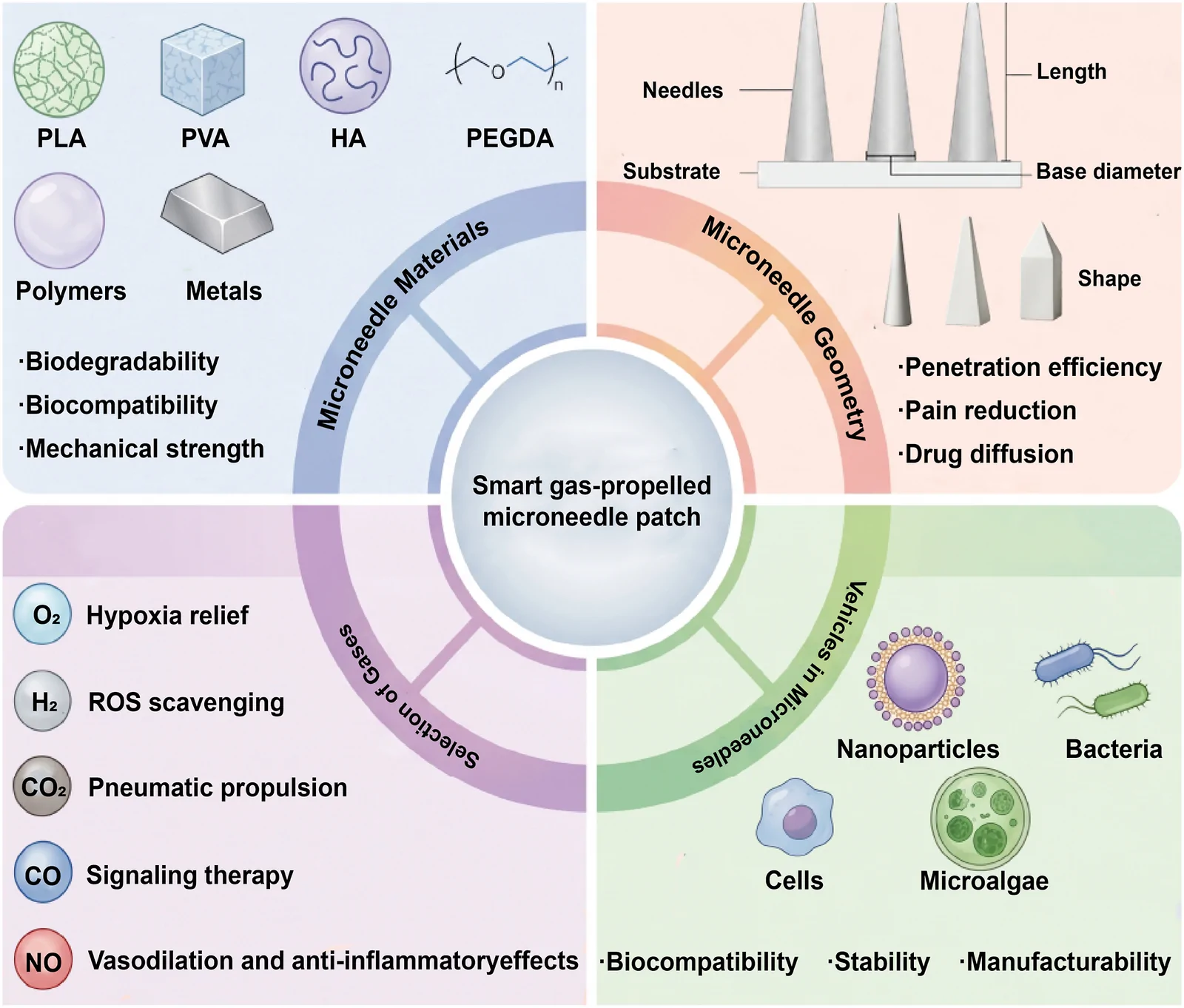
The schematic diagram illustrating the key considerations for microneedle design.

Therefore, factors such as the materials used for the microneedles, geometry, gas selection, and vehicles collectively influence the overall effectiveness of gas-propelled microneedle (Fig. 8).

## 5 Discussion and perspectives

By combining gas therapy with microneedle technology, we can enhance both the safety and efficacy of gas treatments. This approach also facilitates the dissolution and release of drugs beneath the skin, improving the depth, diffusion range, and efficiency of transdermal drug delivery through microneedles.

### 5.1 To perfect the evaluation system

In gas-propelled microneedles, the behavior of gas diffusion and pressure distribution within the skin's microenvironment is crucial. Once the gas is injected into the subcutaneous tissue, the gas molecules start to spread through the tissue in accordance with Fick's laws of diffusion [145]. The skin's porous structure and intricate physiological properties render gas diffusion and pressure application relatively predictable and controllable. By integrating Darcy's Law ($Q=-\frac{kA}{\mu }\frac{\Delta P}{L}$) and Fick's Law ($J=-D\frac{\mathrm{d}C}{\mathrm{d}x}$), we can theoretically develop a mathematical model to analyze gas and drug diffusion, enhancing our understanding of how gas pressure influences drug delivery. This represents a promising avenue for further research in the development of gas-propelled microneedles [146].

For future research, it is essential to explore in greater detail the physical models governing the diffusion of gas through microneedle pores, as well as the models describing the transport of drugs from the outer layer into deeper tissues. Additionally, quantitatively determining the contributions of factors such as gas propulsion force and microneedle length to drug delivery is crucial. This will provide theoretical support for subsequent microneedle-related studies. Moreover, further investigation is needed regarding the diffusion of drugs with varying molecular weights, including high-molecular-weight drugs like proteins and cellular therapies. It is important to assess whether gas-propelled microneedles can provide sufficient propulsion for these substances, thereby expanding their applications in various clinical settings.

### 5.2 To improve biocompatibility and controllability

While some metal materials and endogenous reactive molecules have been employed as fuels for drug delivery, transdermal therapies face significant challenges. The complexity of the body's microenvironment, coupled with the inherent properties of these materials, raises concerns regarding the volume of gas generated, safety issues, and the uncontrollable nature of the reactions involved [69].

To address these challenges, it is crucial to harness effective biological components, such as simple nutrients like glucose. By doing so, we can enhance compatibility with biological systems, leading to more controlled reactions and lower costs [147]. Utilizing naturally occurring substrates not only aligns better with physiological processes but also minimizes potential adverse effects associated with foreign materials. Furthermore, the integration of controllable gas propulsion techniques with microneedle-based transdermal drug delivery methods presents a promising avenue for innovation. For example, Pt nanoparticles and H_2_O_2_ have been employed within the reaction chamber as a built-in fuel source for active and controllable payload delivery [148,149]. Such a combination could improve the precision of drug delivery, enable targeted therapy while ensure patient safety. This interdisciplinary approach could lead to breakthroughs in how we understand and utilize microneedles, potentially revolutionizing drug delivery systems and expanding their applications in clinical settings.

In addition to improving biocompatibility and controllability, comprehensive safety evaluation should also be considered during the design of gas-propelled microneedle systems. Although therapeutic gases are generally regarded as biocompatible at appropriate doses, excessive or rapid gas generation may potentially lead to undesirable local mechanical effects, particularly in loose connective tissues. While no cases of subcutaneous emphysema have been reported in the currently available studies on gas-propelled microneedles, the kinetics and amount of gas generation should nevertheless be carefully optimized to minimize such potential risks. Furthermore, gas-generating systems based on magnesium-, calcium-, or other metal-containing materials inevitably produce inorganic reaction byproducts. Although these materials are generally considered biocompatible, their long-term metabolic fate, clearance efficiency, and potential accumulation following repeated administration remain insufficiently understood. Future studies should therefore incorporate systematic long-term biosafety evaluations, including residue clearance, biodegradation, and chronic toxicity assessments, to facilitate safe clinical translation.

### 5.3 To enhance clinical translational potential

The mass production of microneedle technology faces several challenges that hinder its advancement. These include inadequate quality control standards, such as ISO 13485 (Medical Devices-Quality management systems-Requirements for regulatory purposes) and GMP (Good Manufacturing Practice), which can lead to product quality issues. Regulatory approval processes, like those of the FDA and CE (Conformité Européenne), often reject applications due to insufficient clinical trial data, while differences in regulations can complicate market entry [150]. Additionally, high clinical trial costs and time delays further impede progress. Insufficient intellectual property protection allows competitors to imitate products, and a lack of market education may lead consumers to doubt the safety of new technologies, negatively impacting sales. Moreover, the constantly evolving regulatory environment requires companies to frequently adjust their compliance processes, increasing operational costs [151]. In addition to the general translational challenges encountered by conventional microneedle systems, gas-propelled microneedles introduce several unique considerations. The long-term stability of gas-generating reagents during storage, reproducibility of gas generation kinetics, consistency of propulsion performance, and standardized evaluation of gas-release behavior are all critical factors that may directly influence therapeutic efficacy and regulatory approval. Addressing these issues through optimized formulation design, standardized manufacturing protocols, and robust quality control will be essential for future clinical translation.

Despite these obstacles, collaboration across scientific disciplines, including engineering, materials science, and biomedicine, can help navigate the transition from fundamental research to clinical practice [152]. The treatment regimen for microneedle therapy needs to be tailored to the specific condition of each patient, with careful adjustment of treatment frequency and duration. For chronic conditions, such as diabetes or dermatological diseases, microneedle therapy can be scheduled once a month or once a week, utilizing gas-sustained-release microneedle systems to ensure the gradual and consistent release of medication, thereby reducing the patient's treatment burden [113]. For acute conditions, such as infections or acute inflammation, a higher frequency of treatment may be required, such as several times per week, using gas-fast-release microneedle systems to achieve rapid therapeutic effects [153]. Additionally, the treatment plan should be adjusted based on the patient's responses, including monitoring drug concentration, treatment outcomes, and potential side effects. If necessary, a digital platform can be employed for real-time adjustments to the regimen, optimizing treatment effectiveness and improving patient adherence. Overall, microneedle therapy should prioritize individualization, flexibility, and patient comfort to maximize therapeutic outcomes while minimizing adverse effects [150]. Microneedles offer a convenient alternative to many time-consuming and painful medical procedures, which may motivate a faster transfer to the market. Expanding the applications of microneedles into areas such as theranostics, combining therapy and diagnostics, can lead to significant advancements, enhancing convenience and reducing costs. In recent years, there has been a growing emphasis on personalized medicine, which aims to tailor biomedical decisions to individual patients [154]. To meet this objective, it is crucial to overcome the limitations of a one-size-fits-all approach in microneedle design. Developing new microneedle designs that allow for high flexibility will be essential in creating variants suited for specific applications, aligning with the goals of personalized medicine.

Although no gas-propelled microneedle systems have yet entered clinical trials, the clinical translation of therapeutic gases has progressed steadily in recent years, particularly for hydrogen therapy. Hydrogen inhalation has been investigated in multiple clinical studies for indications including ischemia-reperfusion injury, COVID-19-associated respiratory disorders, metabolic diseases, and neurological disorders, demonstrating favorable safety profiles and encouraging therapeutic potential. These clinical advances support the translational feasibility of hydrogen as a therapeutic gas. In contrast, integrating hydrogen delivery with microneedle-based active transdermal systems remains at the preclinical stage. Future development should therefore focus on establishing standardized manufacturing processes, scalable fabrication, and rigorous clinical evaluation to bridge the gap between proof-of-concept studies and clinical application.

### 5.4 Toward intelligent and precision gas-propelled microneedles

Beyond addressing the current technical challenges, the future development of gas-propelled microneedles is expected to move toward intelligent, programmable, and precision therapeutic platforms rather than simple gas-generating delivery systems. One promising direction is the development of disease-responsive gas-propelled microneedles capable of dynamically regulating gas generation according to local pathological cues, such as pH, reactive oxygen species, enzymes, hypoxia, or inflammatory mediators, thereby enabling spatiotemporally controlled drug delivery. In parallel, multi-gas co-delivery represents an emerging strategy for further expanding the therapeutic functions of gas-propelled microneedles. By integrating gases with complementary biological activities, such as O_2_ with NO or H_2_ with CO, future systems may simultaneously regulate multiple pathological processes within complex disease microenvironments. Such platforms could potentially combine the physical propulsion effect of gas generation with the synergistic or complementary therapeutic effects of multiple gaseous mediators. For example, Zhao et al. [155] engineered a CO_2_ bubble-powered microneedle patch for photothermally regulated NO release to treat biofilm-infected diabetic wound. The system consists of NO donor-loaded nanoparticles co-embedded with NaHCO_3_ in a soluble hyaluronic acid microneedle array. Upon application to the acidic biofilm microenvironment, NaHCO_3_ decomposes to generate CO_2_ bubbles that actively propel nanoparticles deep into the biofilm, while near-infrared irradiation triggered NO release. *In vivo,* the patch effectively eradicated biofilms and promoted collagen deposition and angiogenesis. Asad Ullah et al. [156] also developed a photo-crosslinked GelMA microneedle array capable of the synergistic co-delivery of O_2_ and NO for diabetic wound treatment, in which NO promoted angiogenesis at the wound site, while O_2_ suppressed bacterial infection, thereby facilitating wound healing. The dual-release microneedle array increased fibroblast proliferation by approximately 10% and cell migration by approximately 30% in vitro. In vivo, the patches accelerated wound healing without evident local toxicity or inflammation, while histological and immunohistochemical analyses showed reduced TNF-α and increased TGF-β and IL-10 expression. However, multi-gas systems also introduce additional design challenges, including independent control of gas-generation kinetics, relative dosing, release sequence, and potential interactions between different gases. Therefore, rational selection and coordinated regulation of gas combinations will be essential for developing precise multi-gas therapeutic systems. Another important trend is the integration of gas propulsion with biosensing technologies and wearable electronics to establish closed-loop therapeutic systems that continuously monitor disease biomarkers and automatically adjust drug release. In addition, advances in artificial intelligence, computational modeling, and digital twin technologies may facilitate rational formulation design by predicting gas-generation kinetics, propulsion efficiency, and drug transport behavior, thereby accelerating optimization and reducing experimental costs. Furthermore, integrating gas propulsion with multifunctional biomaterials may enable next-generation theranostic platforms that simultaneously achieve active drug delivery, real-time monitoring, and personalized therapy. Collectively, these interdisciplinary advances are expected to transform gas-propelled microneedles from proof-of-concept delivery systems into versatile precision medicine platforms for a wide range of biomedical applications.

## 6 Conclusion

The development and application of gas-propelled microneedles have attracted increasing interest, holding the potential to significantly transform the healthcare industry. This innovative technology stands out as a promising candidate for enhancing clinical applications in the coming years. However, there is currently a lack of established protocols for the clinical implementation and large-scale production of gas-propelled microneedles. Moreover, several critical challenges remain before gas-propelled microneedles can realize their full clinical potential. These include the incomplete understanding of gas-assisted transport mechanisms, limited control over gas generation kinetics, concerns regarding long-term biosafety and material residues, the lack of standardized evaluation criteria, and insufficient clinical validation. Addressing these challenges will require interdisciplinary collaboration among materials scientists, pharmaceutical researchers, biomedical engineers, and clinicians. Looking forward, the development of intelligent and programmable gas-generating systems, quantitative transport models, disease-specific design strategies, and scalable manufacturing technologies is expected to accelerate the translation of gas-propelled microneedles from laboratory research to practical clinical applications. By integrating active propulsion with the intrinsic therapeutic functions of gaseous mediators, next-generation gas-propelled microneedles hold considerable promise as versatile platforms for precision transdermal drug delivery and localized therapy. To address this gap, it is essential to invest in and promote further research into gas-propelled microneedle, paving the way for their successful integration into healthcare practices.

## CRediT authorship contribution statement

**Zhicheng Xiao:** Writing – review & editing, Writing – original draft. **Fan Jia:** Writing – review & editing, Writing – original draft. **Wentao Wu:** Formal analysis, Data curation. **Hoiian Ieong:** Software, Methodology. **Zeshi Jiang:** Conceptualization. **Siyuan Peng:** Formal analysis. **Chuanbin Wu:** Resources, Project administration, Funding acquisition. **Wenhao Wang:** Supervision. **Xin Pan:** Supervision, Resources, Funding acquisition. **Zhengwei Huang:** Supervision, Funding acquisition.

## Ethics approval and consent to participate

This manuscript is a review article and no animal experiment is included in the manuscript.

## Declaration of competing interest

The authors declare that they have no known competing financial interests or personal relationships that could have appeared to influence the work reported in this paper.
